# Multiplexed ultrasound imaging of gene expression

**DOI:** 10.1038/s41592-025-02825-w

**Published:** 2025-11-18

**Authors:** Nivin N. Nyström, Zhiyang Jin, Marisa E. Bennett, Ruby Zhang, Margaret B. Swift, Mikhail G. Shapiro

**Affiliations:** 1 Division of Chemistry and Chemical Engineering, California Institute of Technology, Pasadena, CA USA; 2 Andrew and Peggy Cherng Department of Medical Engineering, California Institute of Technology, Pasadena, CA USA; 3 Division of Biology and Biological Engineering, California Institute of Technology, Pasadena, CA USA; 4 Howard Hughes Medical Institute, California Institute of Technology, Pasadena, CA USA

**Keywords:** Molecular imaging, Ultrasound

## Abstract

Acoustic reporter genes (ARGs) have enabled imaging of gene expression with ultrasound, which provides high resolution access to deep, optically opaque living tissues. However, unlike their fluorescent counterparts, ARGs have so far been limited to a single ‘sound’, preventing multiplexed imaging of cellular states or populations. Here we use rational protein design and directed evolution to develop two new ARGs that can be distinguished from each other based on their acoustic pressure-response profiles, enabling ‘two-tone’ ultrasound imaging of gene expression. We demonstrate the utility of multiplexed ARGs for delineating bacterial cell species and cell states in vitro, and then apply them towards imaging distinct subpopulations of probiotics in the mouse gastrointestinal tract and of tumor-colonizing bacterial agents in vivo. Just as the first wavelength-shifted derivatives of fluorescent proteins opened a vivid world for optical microscopy, our next-generation acoustic proteins set the stage for a rich symphony of ultrasound signals from living subjects.

## Abstract (web summary)

Ultrasound imaging with acoustic reporter genes has been limited to a single ‘tone’, restricting the types of experiments that can be achieved. This work introduces two acoustic reporter genes that enable multiplexed imaging in vitro and in mice.

## Main

Fluorescent reporter genes have revolutionized the study of living systems. Since the discovery of GFP, an assortment of spectrally orthogonal fluorescent proteins have been developed and are now employed routinely for multiplexed imaging of distinct molecular events within a single field of view^1–3^. Although fluorescence offers unparalleled spatial resolution and sensitivity, it is limited to ex vivo samples or superficial tissues in vivo (≤1 mm) due to light scattering and attenuation^4^.

For deep tissue imaging in living subjects, ultrasound leverages the favorable interactions of acoustic waves with tissue to permit routine imaging at depths of several centimeters and resolutions on the order of 100 µm. In addition, ultrasound imaging offers portability, low cost and high temporal resolution^5^. Recently, acoustic reporter genes (ARGs), which encode air-filled protein nanostructures called gas vesicles (GVs) have been established as ‘acoustic proteins’ for ultrasound imaging of cellular function^6–14^. However, these acoustic analogs of GFP have so far been limited to a ‘single sound’ readout.

We wondered whether it might be possible to generate additional ‘sounds’ of ARGs to enable multiplexed imaging, analogous to the different varieties of fluorescent proteins. This would allow us to measure several variables simultaneously within a single experiment, increasing throughput and data dimensionality, which currently stand as principal bottlenecks in contemporary in vivo imaging methods^15^.

To enable such multiplexing, we sought to engineer ARGs with unique pressure responses. We focused on engineering the secondary structural protein GvpC that scaffolds the GV shell and modifies its stiffness. GVs with a softer shell undergo reversible mechanical buckling under acoustic pressure waves, resulting in nonlinear ultrasound contrast^16–18^. We hypothesized that by engineering GvpC we could tune the buckling threshold of the GVs, thereby producing variants with different pressure responses, allowing us to tell them apart by imaging with a series of suitable pressures. To test this hypothesis, we designed a high-throughput in cellulo acoustic screen and used it to engineer and evolve two next-generation ARGs, which we call bARG_560_ and bARG_710_, for multiplexed imaging of gene expression on ultrasound.

To demonstrate the utility of these ARGs, we introduced them into probiotic microorganisms, allowing us to delineate two species—*Salmonella typhimurium SL1344* (Stm) and *Escherichia coli Nissle 1917* (EcN)—within a single field of view. Unlike previous attempts at acoustic multiplexing^6,16^, distinguishing bARG_560_ from bARG_710_ did not require irreversibly collapsing the GVs and thereby destroying their ultrasound contrast. We also incorporated bARG_560_ and bARG_710_ into a genetic state-switch circuit to visualize two cellular states with ultrasound. Finally, we employed these new ARGs for multiplexed, deep tissue, high spatial resolution imaging in live mice, demonstrating the capacity to distinguish between microbial species in the gastrointestinal (GI) tract and track co-colonization of tumors by distinct populations of tumor-homing bacteria.

## Results

### Removal of gvpC and construct optimization results in ARGs with a low pressure threshold and strong nonlinear contrast

**Fig. 1:**
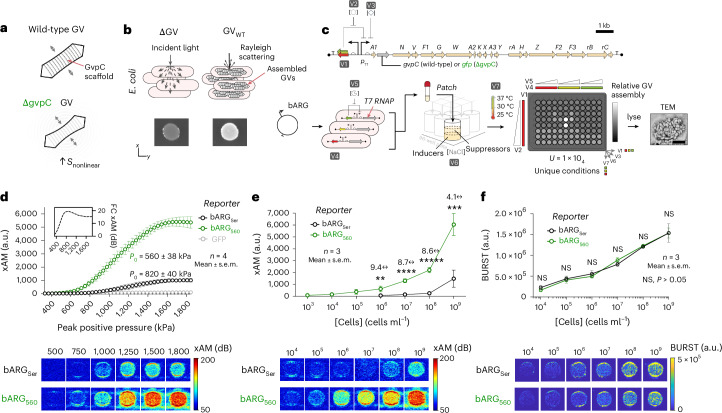
In cellulo assembly screening for an acoustic reporter gene with increased nonlinearity. **a**, Top: wild-type GV nanostructure with its native GvpC protein scaffold. Bottom: GVs lacking GvpC exhibit an increased amplitude of reversible buckling resulting in stronger nonlinear ultrasound signal. **b**, GVs assembled within *E.* *coli* scatter light, resulting in patches with increased opacity. **c**, High-throughput assembly screen to identify circuit conditions for in cellulo assembly of GVs with a *gvpC* deletion. Top: annotated *Serratia* sp. 39006 GV operon, with *gvpC* (bARG_Ser_), or with *gvpC* replaced by *gfp* (bARG_560_). Bottom, from left to right: plasmids were transformed into *E.* *coli* and grown in liquid culture before being patched on dual-layer 96-well agar plates. Plates were assessed for GV assembly via patch opacity. TEM, transmission electron microscopy. Scale bar, 200 nm. **d**, Nonlinear acoustic signals measured at variable pressures from *E.* *coli* encoding bARG_Ser_ or bARG_560_ (mean ± s.e.m.; *n* = 4). xAM, X-wave amplitude modulation signal. Inset: nonlinear signal fold-change (FC) across pressures between bARG_Ser_ and bARG_560_ in decibels (dB). **e**, Nonlinear acoustic signals of *E.* *coli* encoding bARG_Ser_ or bARG_560_ at variable cell concentrations. Multiple unpaired *t*-tests were performed for statistical analysis at each cell concentration, without *P* value correction for multiple comparisons (mean ± s.e.m.; *n* = 3; **P = 0.01, ***P = 0.003, ****P = 0.0001, *****P = 0.00003). **f**, Buckling-independent quantification of GVs in *E.* *coli* encoding bARG_Ser_ or bARG_560_ at variable cell concentrations. Multiple unpaired *t*-tests were performed for statistical analysis at each cell concentration, without *P* value correction for multiple comparisons (mean ± s.e.m.; *n* = 3, *P* > 0.05). Source data

To develop multiplexed ARGs, we started with the most recently developed bacterial ARG system derived from *Serratia* sp. 39006 (bARG_Ser_), which has been used to enable sensitive nonlinear imaging of genetically modified bacteria in vitro and in vivo^10^. Alongside other GV-forming genes, bARG_Ser_ encodes GvpC—an alpha-helical protein that binds to the GV shell to increase its stiffness. To generate ARGs with more permissive shell buckling, and therefore nonlinear contrast at lower acoustic pressures, we first deleted *gvpC* from bARG_Ser_ and replaced it with *gfp* (Fig. 1a), and used a high-throughput optical assay based on the optical scattering property of assembled GVs in multiwell bacterial patches to optimize the genetic construct and its expression conditions (Fig. 1b,c). Screening ~10^4^ unique conditions (Fig. 1c) yielded a Δ*gvpC* construct with highly efficient expression and minimal burden. In this construct, the GV operon is transcribed by an isopropyl-β-d-thiogalactoside (IPTG)-dependent T7 RNA polymerase expressed under l-arabinose control (Supplementary Table 1).

To measure the resulting acoustic contrast, we loaded cells into agarose phantoms and acquired nonlinear ultrasound images at transmit pressures ranging from 0.3 to 1.8 MPa using a cross-propagating wave (x-wave) amplitude modulation pulse sequence^19^ (xAM; *n* = 4). With our new construct, we observed a decreased nonlinear threshold (*P*_o_ = 560 ± 38 kPa) and an increased nonlinear signal yield (*S*_max_ = 5,900 ± 660 a.u.) relative to the wild-type operon (*P*_o_ = 820 ± 40 kPa, *S*_max_ = 1,000 ± 130 a.u.; Fig. 1d). We named this new construct bARG_560_ on the basis of its nonlinear pressure threshold. With respect to imaging sensitivity, bARG_560_ exhibited fourfold to ninefold higher nonlinear signals relative to bARG_Ser_ across a range of cell concentrations (Fig. 1e), whereas overall GV production, as measured using the buckling-independent BURST pulse sequence^20^, was not significantly different between the two populations (*n* = 3; *P* > 0.05; Fig. 1f).

### Directed evolution of GvpC results in ARGs with shifted pressure-response profiles

**Fig. 2:**
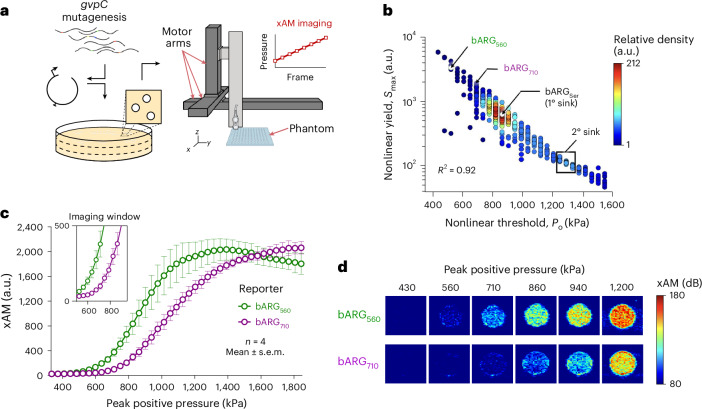
Directed evolution of acoustic reporter genes for pressure-domain multiplexing. **a**, An error-prone PCR library of *gvpC* was cloned into a backbone plasmid containing the bARG_560_ operon, transformed into *E.* *coli* and then plated. Colonies were arrayed, grown and induced for expression, after which they were loaded into agarose phantoms for nonlinear acoustic measurements on a custom-built motorized ultrasound system. **b**, Acoustic map of the *gvpC* mutagenesis library. *P*_o_, nonlinear pressure threshold, *N* = 879. *S*_max_, maximum nonlinear yield. Each point represents a mutant (*N* = 879). Color scale represents the relative density of mutants on the plot. Controls include bARG_Ser_ (white circle) and bARG_560_ (green circle). **c**, Nonlinear acoustic signals measured at increasing pressures from *E.* *coli* encoding bARG_560_ and bARG_710_ (mean ± s.e.m.; *n* = 4). xAM, inset: nonlinear imaging window, capped at the collapse pressure of bARG_560_. **d**, Nonlinear images of *E.* *coli* encoding bARG_560_ or bARG_710_ acquired across increasing pressures. Source data

**Extended Data Fig. 1:**
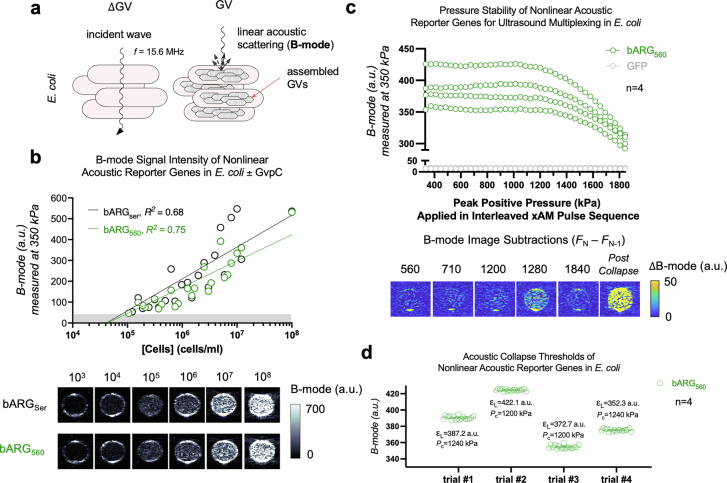
Determination of the acoustic collapse pressure of bARG_560_. (**a**) *E. coli* inherently produce minimal linear scattering on ultrasound, but GVs produced within *E. coli* can operate as linear scattering agents at low acoustic pressures on B-mode images. (**b**) B-mode signal intensity (a.u.) at 350 kPa measured across increasing concentrations of *E. coli* (10^3^–10^8^ cells ml^−1^, n=3) encoding bARG_Ser_ (*black*) or bARG_560_ (*green*), and representative B-mode images of each. (**c**) B-mode signal intensity (a.u.) at 350 kPa of *E. coli* encoding bARG_560_ (n=4) following X-wave amplitude modulation (xAM) pulse sequence imaging at increasing pressures (0.3–1.8 MPa, ∆*P*_N-(N-1)_ ≅ 40 kPa). B-mode image subtractions (a.u.). The ‘Post Collapse’ image represents the subtraction between a B-mode image acquired after applying the maximum xAM pulse sequence pressure (1.8 MPa) and a B-mode image acquired after applying a parabolic B-mode collapse pulse. (**d**) To determine the acoustic collapse pressure of bARG_560_ nanostructures, B-mode signal intensity at 350 kPa was averaged across 10 acquisitions for each sample, and the lower limit of the 95% confidence interval (ε_L_) was calculated for each trial (n=4). The collapse pressure was defined as the threshold at which B-mode signal intensity first fell below the lower limit of the 95% confidence interval. Source data

To engineer a second ARG with strong nonlinear scattering and a pressure threshold distinctly higher than that of bARG_560_, we performed a directed evolution screen of GvpC (Fig. 2a). Our target pressure profile would result a nonlinear signal threshold upshifted by 100 kPa or more relative to bARG_560_, while producing strong contrast below the collapse pressure of bARG_560_ (*P*_c_ = 1,220 ± 34 kPa; Extended Data Fig. 1) to enable continuous multiplexed imaging without destroying GVs. To achieve these specifications, we ran a directed evolution campaign (Fig. 2a). We used error-prone PCR to generate a pooled mutagenesis library of *gvpC*, induced *E.* *coli* colonies containing the resulting variants on solid agar and loaded samples into 96-well agarose phantoms for acoustic measurements on a custom-built motorized ultrasound system (Fig. 2a). To organize our library dataset, we first set a linear scattering (B-mode) intensity threshold (*S*_B_ ≥ 175 a.u.) to filter out samples with insufficient GV expression, and plotted all remaining mutants according to their nonlinear signal threshold (*P*_o_) and maximum nonlinear signal yield (*S*_max_; Fig. 2b). Altogether, we obtained data from 879 variants.

We observed a strong negative logarithmic correlation (*R*^2^ = 0.92) between the nonlinear threshold and maximum nonlinear yield of the variants in our library, which was not unexpected: *gvpC* sequences that confer greater rigidity to the GV shell, and require larger force to initiate buckling, are also likely to limit the amplitude of GV deformation, and the resulting sound scattering, after buckling. bARG_560_, included as a control, sits near the upper left corner of the acoustic map (*P*_o_ = 560 kPa, *S*_max_ = 3,400 a.u.). We observed two acoustic ‘sinks’—regions of the plot that contained high concentrations of sequences. The deeper sink was centered on the parent *gvpC* (bARG_Ser_; *P*_o_ = 860 kPa; *S*_max_ = 1,190 a.u.) from which all these mutants were derived. The second and shallower sink contained sequences with relatively high nonlinear thresholds and low nonlinear signal within the tested pressure range.

**Extended Data Fig. 2:**
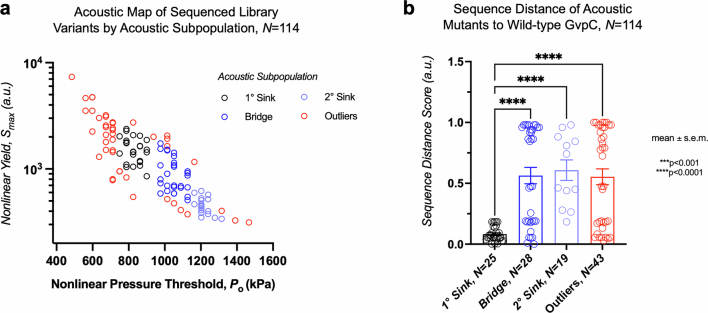
Sequence distance scores of mutants to wild-type *gvpC* by acoustic subpopulation. (**a**) Acoustic map of sequenced library variants according to their acoustic subpopulation: 1° acoustic sink centered on the wild-type *gvpC* sequence (*black)*, 2° acoustic sink (*purple*), the bridge between them (*blue*), and any mutants that fell outside of these regions (*red*). Refer to Fig. 2b for the parent acoustic map containing all variants (sequenced and not sequenced), colored by the relative density of mutants. (**b**) Average sequence distance scores (mean ± s.e.m., a.u.) of acoustic subpopulations following Needleman-Wunsch alignment to wild-type *gvpC*. ***p<0.001, ****p<0.0001. Source data

To gain a deeper understanding of our sequence landscape and how it relates to our acoustic map, we sequenced a subset of mutants from different acoustic regions (primary (1°) sink, secondary (2°) sink, the bridge between them and outliers that resided outside of these regions), and performed pairwise Needleman–Wunsch protein sequence alignments of GvpC against the wild-type parent (Extended Data Fig. 2). Not surprisingly, we found that sequences within the 1° acoustic sink exhibited the lowest sequence distance scores (*D*_1_ = 0.088 ± 0.01; *N* = 25), signifying that single mutations were generally conservative with respect to acoustic properties. Meanwhile, we saw a significant increase in sequence distance in variants in the 2° sink (*D*_2_ = 0.61 ± 0.07; *N* = 19) and the bridge between the sinks (*D*_b_ = 0.50 ± 0.08; *N* = 28), and in that of acoustic outliers that fell outside of these regions on the map (*D*_4_ = 0.53 ± 0.06; *N* = 43).

**Extended Data Fig. 3:**
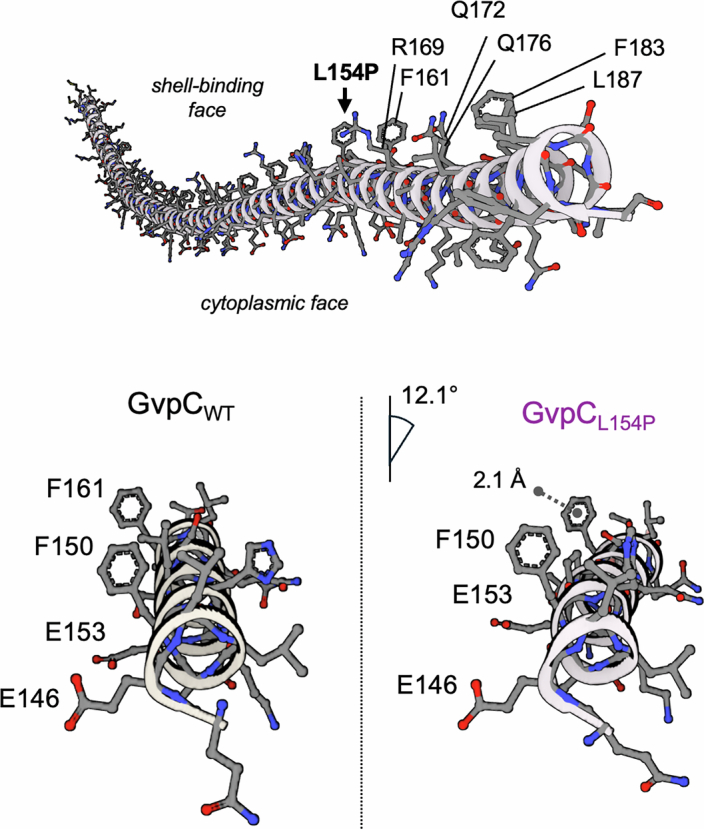
AlphaFold structural prediction of GvpC_L154P_. *Top:* Ball-and-stick model of the AlphaFold structural prediction of GvpC_L154P_ with GV-binding residues labelled downstream of the L154P structural kink. *Bottom:* wild-type gvpC structure (*left*) and GvpC_L154P_ (*right*) spanning from Q145 to T165, with angle deviation (12.1°) and F161 distance differential (2.1 Å) denoted.

After evaluating several outlier variants that met our design specifications for pressure-domain multiplexing, we selected a mutant containing an L154P mutation that conferred a nonlinear signal threshold of 710 ± 32 kPa and a nonlinear yield comparable to that of bARG_560_ within the ‘nondestructive imaging window’ below 1,200 kPa (Fig. 2c). An AlphaFold^21^ structural prediction of GvpC_L154P_ compared to the parent protein suggests that a proline-imposed kink in the alpha helix may result in reduced contact with the GV shell (Supplementary Note 1 and Extended Data Fig. 3). We call this evolved construct bARG_710_, according to its nonlinear threshold in kilopascals.

### Multiplexed ultrasound can distinguish two cell types or cell states

After obtaining two ARGs with acoustic properties compatible with multiplexing, we tested their ability to render two cell types or two cell states visible to ultrasound in the same sample. The ability to delineate distinct cell populations in deep tissues of living subjects at high spatial resolution would be useful across many applications, including cell-based diagnostics and therapies^22,23^. As a first step in this direction, we engineered two widely studied probiotic microorganisms—attenuated Stm^24^ and EcN^25^—to express bARG_560_ and bARG_710_, respectively. These probiotic strains are popular in the development of cell-based diagnostics and therapies due to their ability to populate the GI tract, colonize the hypoxic core of tumors, sense local biomarkers and produce therapeutic payloads, and have already entered human clinical trials^26–28^.

**Extended Data Fig. 4:**
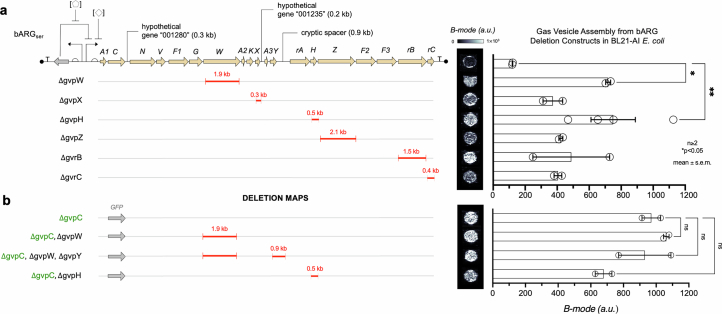
GV assembly screening of gene deletion constructs in BL21-AI *E. coli.* (**a**) Construct and single gene deletion maps based on the bARG_Ser_ construct. B-mode signal intensity (a.u.) at 350 kPa of bARG_Ser_ and single gene deletion constructs measured from 10^6^ cells ml^−1^ in BL21-AI *E. coli* (mean ± s.e.m., n≥2). (**b**) Multi-gene deletion maps based on the bARG_560_ construct. B-mode signal intensity (a.u.) at 350 kPa of bARG_560_ and multi-gene deletion constructs measured from 10^9^ cells ml^−1^ in BL21-AI *E. coli* (mean ± s.e.m., n≥2). Source data

**Extended Data Fig. 5:**
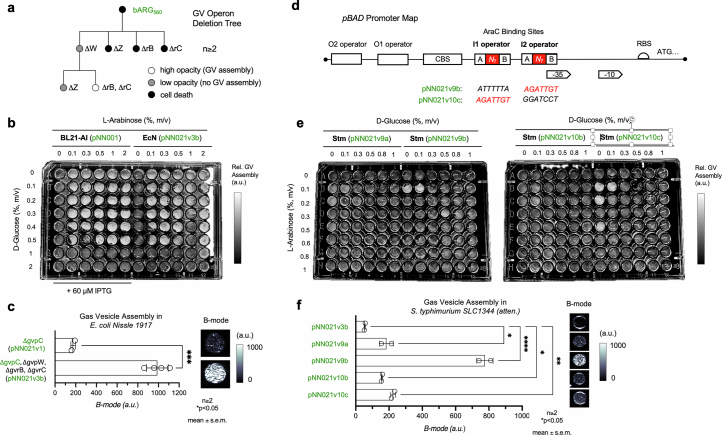
Engineering GV assembly constructs for probiotic microorganisms. (**a**) Synthetic evolutionary tree of bARG_560_ in *E. coli Nissle 1917* (EcN). Each construct was transformed into EcN and tested across a range of induction conditions, which resulted in either cell death upon induction (*black*), or cell survival with (*white*) or without (*grey*) GV assembly (n≥2). (**b**) Representative 96-well patch plate of BL21-AI *E. coli* transformed with bARG_560_ in a T7-circuit (pNN001; *left half of plate*) and *E. coli Nissle 1917* (EcN) transformed with bARG_560_ that includes deletions of gvpW, gvrB, and gvrC (pNN021v3b; *right half of plate*), induced for GV expression on dual-layer agar containing variable concentrations of L-arabinose (0–2%, m/v) and D-glucose (0–2%, m/v). Non-variable conditions include 60 µM IPTG for BL21-AI *E. coli*, 37 °C incubation, and 5 g/L NaCl. (**c**) B-mode signal intensity (a.u.) at 350 kPa of *E. coli Nissle 1917* transformed with pNN021v1 (pBAD, *gfp*^+^, Δg*vpC*) or pNN021v3b (pBAD, *gfp*^+^, Δg*vpC*, Δg*vpW*, Δg*vrB*, Δg*vrC;* mean ± s.e.m., n≥2). (**d**) Promoter map of pBAD. AraC binding sites include the O2, I1, and I2 operators. CBS, cAMP receptor protein (CRP) binding site. RNA polymerase binding sites, −35 and −10 elements. RBS, ribosome binding site. ATG, start codon. The 7 nucleotides within the I1 and I2 operators, internal to the A and B boxes, were targeted for random mutagenesis in attenuated *Salmonella typhimurium SL1344* (Stm). Successful mutant sequences from the pooled library that exhibited GV assembly (pNN021v09b, pNN021v10c) are included: wild-type sequence in black, mutant sequence in red. (**e**) Representative 96-well patch plate of select Stm colonies from a pooled library transformation of I1 and/or I2 mutant constructs, induced for GV expression on dual-layer agar containing variable concentrations of L-arabinose (0–1%, m/v) and D-glucose (0–1%, m/v). pNN021v09b and pNN021v10c exhibited increased opacity, indicative of GV assembly. (**f**) B-mode signal intensity (a.u.) at 350 kPa of Stm transformed with pNN021v3b (pBAD, *gfp*^+^, Δg*vpC*, Δg*vpW*, Δg*vrB*, Δg*vrC*) and various pBAD mutant constructs derived from pNN021v3b (mean ± s.e.m., n≥2). Source data

To implement our new ARGs in these two species, we optimized the corresponding genetic constructs extensively by testing several *gvp* gene deletions and promoter sequences (Supplementary Note 2 and Extended Data Figs. 4 and 5) on an axe/txe stabilized plasmid^29^. For bARG_710_, we placed *gvpC*_L154P_ onto its own operon under regulation of a constitutive promoter derived from *pTac*^30^, and co-expressing a red fluorescence reporter gene, *dsRed2*.

**Fig. 3:**
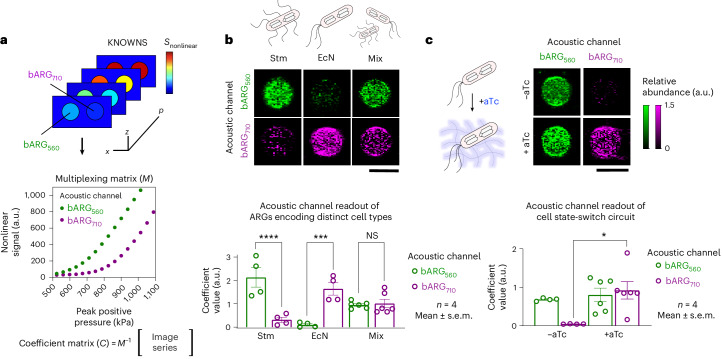
Multiplexed imaging of cell types and cell states on ultrasound with acoustic reporter genes. **a**, A multiplexing matrix *M* was produced by measuring nonlinear signals from pure populations of cells encoding bARG_560_ or bARG_710_ across the pressure domain. *M* was then used for pixelwise linear unmixing of input images containing pure or mixed reporter samples, solving for the coefficient matrix *C*. **b**, Representative coefficient matrix images, or ‘acoustic channel’ images, of pure or mixed populations of attenuated Stm encoding bARG_560_ and EcN encoding bARG_710_. Mean coefficient values (mean ± s.e.m., *n* = 4, a.u.) of acoustic channel images from pure or mixed populations of Stm encoding bARG_560_ and EcN encoding bARG_710_. Ordinary one-way ANOVA was performed for statistical analysis (****P < 0.00001, ***P = 0.0007, NS = 0.9). Scale bar, 2 mm. **c**, Representative acoustic channel images of cells encoding the tetracycline-responsive bARG_560_/bARG_710_ state-switch circuit, induced for expression in the absence (−) or presence (+) of anyhydrotetracycline (aTc). Mean coefficient values (mean ± s.e.m., *n* = 4, a.u.) of acoustic channel images from cells induced for expression in the absence or presence of aTc. Ordinary one-way ANOVA was performed for statistical analysis (*P = 0.02). Scale bar, 2 mm. Source data

To enable the acquisition of multiplexed ultrasound images, we generated a multiplexing matrix (*M*) by measuring nonlinear signals from pure populations of bacteria encoding either bARG_560_ or bARG_710_ across the pressure domain, up to the collapse pressure of bARG_560_ (Supplementary Table 3). We then used *M* for pixelwise linear unmixing of images containing our samples, solving for the coefficient matrix (*C*) of our images via linear algebra (Fig. 3a). *C* contains the estimated contributions of our two acoustic reporters to the signal in each pixel, allowing us to create spatial maps of each reporter gene, which we refer to as our ‘acoustic channels.’

**Extended Data Fig. 6:**
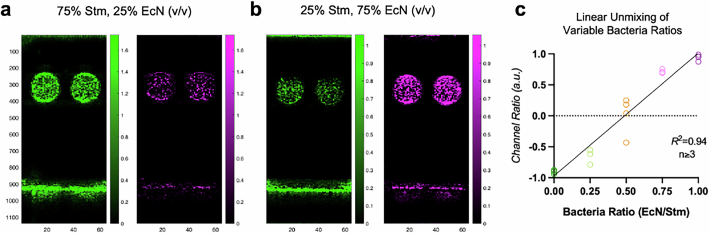
Linear Unmixing of Variable Bacteria Ratios. (**a**) Acoustic channel images of attenuated *S. typhimurium SL1344* (Stm) encoding bARG_560_ and *E. coli Nissle 1917* (EcN) encoding bARG_710_, in a 75:25 volume ratio mixture, and (**b**) in a 25:75 volume ratio mixture. Green channel: bARG_560_. Magenta channel: bARG_710_. (**c**) Normalized difference of the acoustic channels of samples containing different ratios of attenuated *S. typhimurium SL1344* (Stm) encoding bARG_560_ and *E. coli Nissle 1917* (EcN) encoding bARG_710_. Source data

As expected, in pure samples of each type, we observed signals almost entirely on the bARG_560_ channel in images of Stm encoding bARG_560_ (*S*_Stm/560_ = 2.1 ± 0.4 a.u., *S*_Stm/710_ = 0.31 ± 0.1 a.u.; *P* < 0.0001) and the reverse relationship for images of EcN encoding bARG_710_ (*S*_EcN/560_ = 0.099 ± 0.05 a.u., *S*_EcN/710_ = 1.64 ± 0.3 a.u.; *P* = 0.0007). In images of equal-volume mixtures of Stm and EcN, we observed similar signals from the two channels (*S*_mix/560_ = 0.95 ± 0.06 a.u., *S*_mix/710_ = 1.01 ± 0.2 a.u.; Fig. 3b). We tested additional volume ratios of bacteria mixtures and found a linear relationship (*R*^2^ = 0.94) to the acoustic channel ratio (Extended Data Fig. 6).

**Extended Data Fig. 7:**
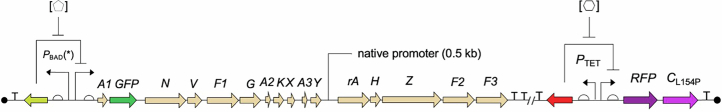
The minimized bARG_560_ operon and the tetracycline-responsive gvpC_L154P_ operon for cell state imaging. The chemical inducer used for the *pBAD*(*) promoter is L-arabinose and the chemical inducer for the *pTET* promoter is anhydrous tetracycline (aTc).

In addition to multiplexing separate cell populations, we wanted to enable the imaging of distinct cell states in a single population, for example ones triggered by an environmental cue. As proof of concept, we built a construct which by default expresses the genes encoding bARG_560_, that is the base GV, but switches to bARG_710_ in response to tetracycline (aTc) by expressing *gvpC*_L154P_ (Extended Data Fig. 7). When we expressed this circuit in EcN cells and imaged them with ultrasound, we found that addition of aTc led to a large increase in signal on the bARG_710_ channel (*S*_−aTc/710_ = 0.042 ± 0.002 a.u., *S*_+aTc/710_ = 0.92 ± 0.2 a.u.; *P* = 0.02; Fig. 3b).

### Multiplexed ARGs enable the imaging of distinct cell populations in the GI tract

**Fig. 4:**
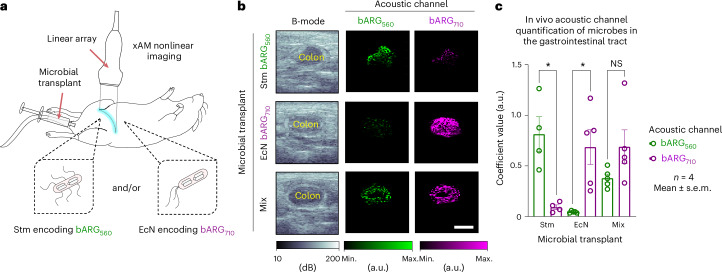
Imaging distinct microbial cell populations in guts of living mice on ultrasound using nonlinear acoustic reporter genes. **a**, Microbial transplant model. Attenuated Stm encoding bARG_560_ and EcN encoding bARG_710_ were transplanted into the GI system of mice. B-mode imaging and xAM nonlinear imaging were performed across the pressure domain, followed by a high-pressure collapse pulse. **b**, Representative B-mode images, acoustic channel images and merged overlay. Scale bar, 2 mm. **c**, Mean coefficient values (mean ± s.e.m., *n* = 4, a.u.) of acoustic channel images of the GI lumen of mice that received microbial transplants of Stm encoding bARG_560_ and/or EcN encoding bARG_710_. Ordinary two-way ANOVA was performed for statistical analysis (*P = 0.02, for both comparisons; NS = 0.5). Max., maximum; min., minimum. Source data

To provide a demonstration of multiplexed ultrasound imaging in the context of living tissue, we imaged our two ARG-expressing bacterial species (Stm-bARG_560_ and EcN-bARG_710_) in the GI tract of live mice. To have precise control over their in vivo distribution, we transplanted these cells into the mouse colon using hydrogel injection. This model has served as a controlled technical proving-ground in previous studies of reporter genes and biosensors for ultrasound^6,31^. Here we transplanted the cells either as pure populations or as mixed communities (Fig. 4a). We used B-mode imaging to locate a cross-section of the GI lumen based on soft tissue contrast before acquiring nonlinear images across the pressure domain. We then performed linear unmixing on the pressure-domain image datasets to generate two coefficient matrices for each mouse, representing each acoustic channel (Fig. 4b).

We saw that mice transplanted with Stm-bARG_560_ generated significantly greater mean coefficient values in the bARG_560_ channel (*S*_Stm/560_ = 0.81 ± 0.2 a.u.) than the bARG_710_ channel (*S*_Stm/710_ = 0.091 ± 0.02 a.u.; *P* = 0.02, *n* = 4). Similarly, we saw that mice transplanted with EcN-bARG_710_ generated significantly greater mean coefficient values in the bARG_710_ channel (*S*_EcN/710_ = 0.69 ± 0.2 a.u.) than in the bARG_560_ channel (*S*_EcN/560_ = 0.044 ± 0.004 a.u., *P* = 0.02, *n* = 4). Finally, we observed significantly increased mean coefficient values in both the bARG_560_ (*S*_mix/560_ = 0.38 ± 0.04 a.u.) and bARG_710_ (*S*_mix/710_ = 0.69 ± 0.2 a.u.) channels from GI images of mice that harbored both microbial populations, relative to signals from negative acoustic channels in control mice (Fig. 4c). These experiments validated that, despite the greater aberration, attenuation and background contrast of living tissues, pressure-based acoustic multiplexing successfully distinguishes two ‘sounds’ of ARGs.

### Multiplexed ARGs enable the imaging of tumor homing by two distinct tumor-colonizing bacteria

Having provided a technical demonstration if in vivo multiplexed imaging in the GI tract, we sought to demonstrate the utility of acoustic multiplexing in a translationally relevant application by imaging two tumor-colonizing bacterial species in the same therapy recipient. Recent research has established that tumors are colonized naturally by bacterial species^32,33^, and that certain strains of bacteria can be used as tumor-colonizing therapeutic agents due to their ability to thrive in the immunosuppressed, hypoxic, necrotic cores of tumors and be engineered to produce oncolytic or immunostimulatory biologics to induce remission^26,28^. In this paradigm, ultrasound can offer the ability to track the successful engraftment of a probiotic cell therapy at its target, whereas multiplexing could provide a way to track successively administered doses, coadministered variants, or conditional cellular states.

**Fig. 5:**
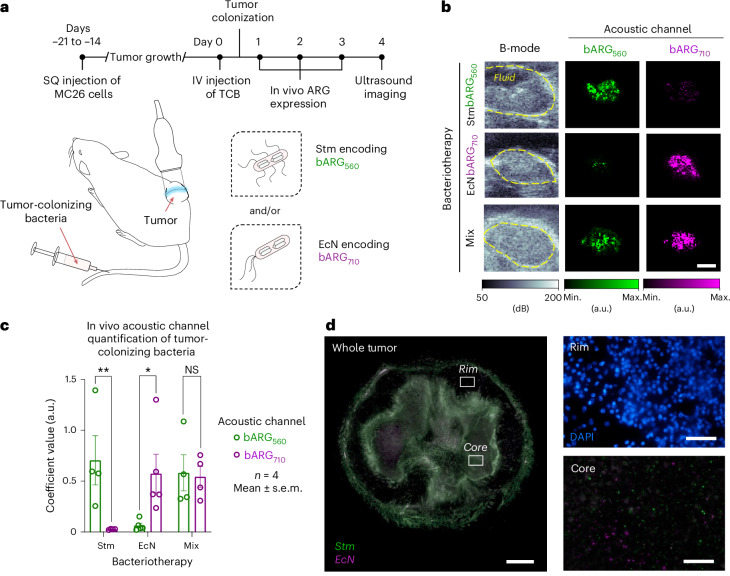
Multiplexed ultrasound imaging of tumor colonization by single- or dual-species probiotic agents. **a**, Mice received subcutaneous injections of MC26 tumor cells into their right hind flanks. Once tumors grew, a dose of tumor-colonizing bacteria, consisting of either attenuated Stm encoding bARG_560_ and/or EcN encoding bARG_710_, was injected intravenously and given 24 h to colonize the tumor. The following day, and every 24 h for the following 2 days, mice were injected intraperitoneally with l-arabinose to induce GV expression in situ. B-mode images and xAM images across the pressure domain were then acquired to image the distribution of each species. **b**, Representative B-mode images and nonlinear bARG_560_ (green) and bARG_710_ (magenta) acoustic channel images of mice receiving different species or mixed populations of tumor-colonizing bacteria. Scale bar, 2 mm. **c**, Mean coefficient matrix values (mean ± s.e.m., *n* = 4, a.u.) of bARG_560_ (green) and bARG_710_ (magenta) acoustic channel images of tumors from mice receiving tumor-colonizing bacteria communities consisting of Stm encoding bARG_560_ and/or EcN encoding bARG_710_. Ordinary two-way ANOVA was performed for statistical analysis (**P = 0.006, *P = 0.01, NS = 0.8). **d**, Left: whole-tumor fluorescence image overlay of GFP (green), and dsRed2 (magenta) channels from a representative mouse that received a 1:1 species mixture (v:v) of attenuated Stm encoding bARG_560_ (green) and EcN encoding bARG_710_ (magenta). White regions represent overlays of both species. Scale bar, 1 mm. Right: high-magnification fluorescence images of the tumor core and the tumor rim. Scale bar, 100 µm. Source data

To provide a proof of concept for multiplexed cell therapy imaging in tumors, we implanted murine colon cancer cells into the right flanks of mice and allowed the tumors to grow for 14–21 days before injecting intravenously pure or mixed populations of Stm-bARG_560_ and EcN-bARG_710_ (Fig. 5a). We induced GV expression for 3 days, starting 24 h after injecting the bacteria, and performed ultrasound imaging on the fourth day. We observed dominant signal from the bARG_560_ channel compared to the bARG_710_ channel in tumors of mice injected with Stm (*S*_Stm/560_ = 0.71 ± 0.2 a.u., *S*_Stm/710_ = 0.026 ± 0.002 a.u.; *P* = 0.006, *n* = 4) and significantly higher signal from the bARG_710_ channel relative to the bARG_560_ channel in tumors of mice injected with EcN (*S*_EcN/560_ = 0.063 ± 0.02 a.u., *S*_EcN/710_ = 0.58 ± 0.2 a.u.; *P* = 0.01, *n* = 5).

**Extended Data Fig. 8:**
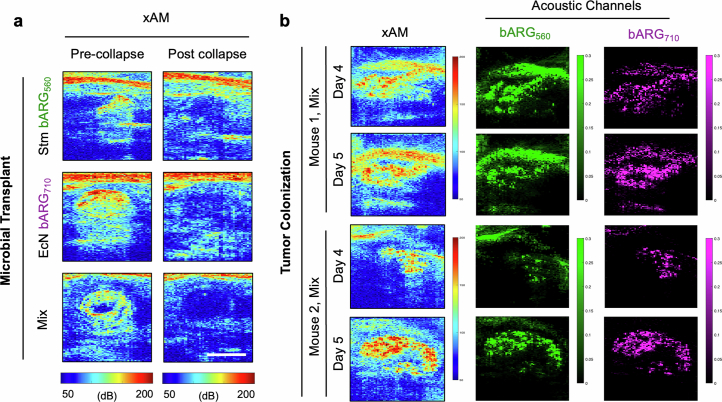
Linear Unmixing of Bacteria in the Gastrointestinal Tract and of Tumor-homing Bacteria. (**a**) Nonlinear signal validation in the gastrointestinal tract of live mice. Representative X-wave amplitude modulation (xAM) images at ~1000 kPa of the gastrointestinal system of mice transplanted with attenuated *S. typhimurium SL1344* (Stm) encoding bARG_560_ and/or *E. coli Nissle 1917* (EcN) encoding bARG_710_, before and after application of a high-pressure collapse pulse. Decibels, dB. Scale bar, 2 mm. (**b**) Longitudinal imaging of tumor colonizing bacteria populations. Representative X-wave amplitude modulation (xAM) images at ~1000 kPa of tumors of two mice that were injected with mixtures of attenuated *S. typhimurium SL1344* (Stm) encoding bARG_560_ and/or *E. coli Nissle 1917* (EcN) encoding bARG_710_, and deconvoluted Acoustic Channel images of each timepoint. *Green channel*: bARG_560_. *Magenta channel*: bARG_710_.

When we administered a 1:1 mixture of Stm and EcN, we observed comparable levels of signals from each acoustic channel (*S*_mix/560_ = 0.58 ± 0.2 a.u., *S*_mix/710_ = 0.54 ± 0.1 a.u.; *P* = 0.85, *n* = 4; Fig. 5b,c), confirming the ability to distinguish pure from mixed colonization scenarios. We were able to image a subset of mice again 24 h following the first imaging scan, thereby suggesting that longitudinal imaging is feasible, although fiducial markers would be very useful in countering differences in probe orientations between scans (Extended Data Fig. 8). To validate that Stm and EcN both colonized the tumor in mice that received mixed population doses, and that interspecies competition did not result in single-strain dominance, we performed tumor histology. In agreement with our ultrasound images, we saw co-colonization of the tumor core by both bacterial species, and validated in vivo induction of both bARG_560_ and bARG_710_ operons through visualization of their fluorescent markers within the same tumor (Fig. 5d).

## Discussion

This work establishes multiplexed ultrasound imaging of gene expression using two next-generation ARGs. We anticipate that the ability to track several cell types or states simultaneously within individual living subjects will enable more comprehensive study of complex biological systems. Although other imaging modalities have already benefited from multiplexing^34–36^, ultrasound can now also harness this capability. In the process of engineering bARG_560_ and bARG_710_, we established bespoke methods for ARG engineering that may be extended to related applications. For example, the high-throughput GV assembly optimization methods we used to enable bARG_560_ expression could help domesticate GV-encoding gene clusters from other species or adapt ARGs to additional microbial species such as native members of the gut microbiome.

**Extended Data Fig. 9:**
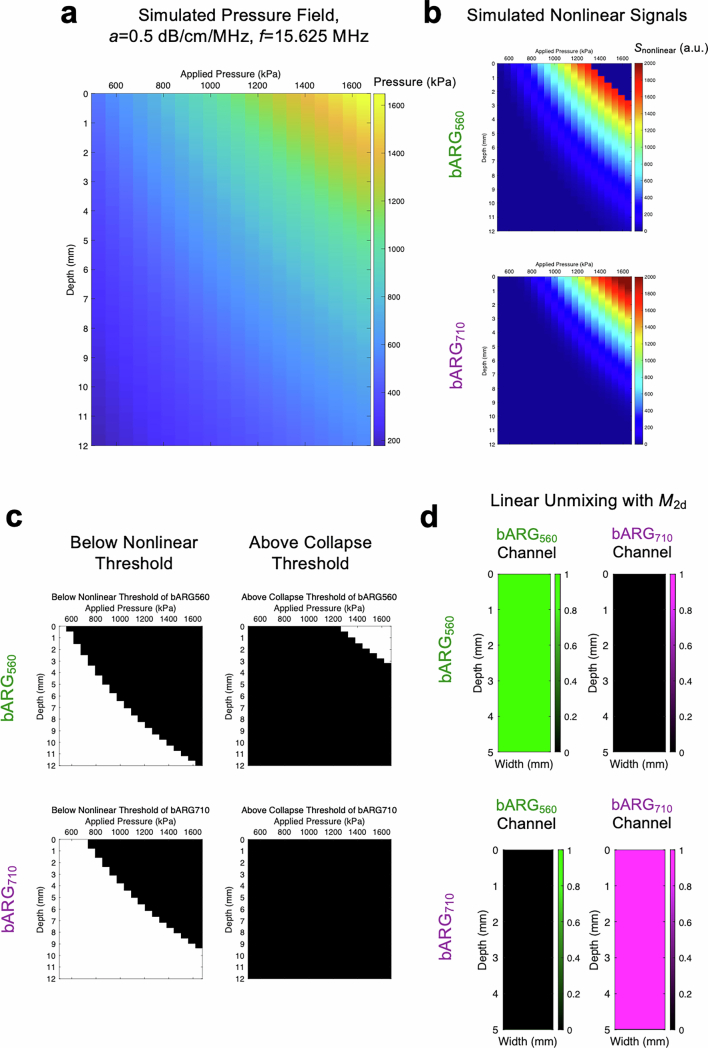
Acoustic Unmixing with Depth Correction. (**a**) Simulated acoustic pressure field with 0.5 dB/cm/MHz attenuation, at a frequency of 15.625 MHz, at increasing applied pressures. (**b**) Simulated nonlinear signals of acoustic reporters placed homogenously across the depth of the field-of-view. (**c**) Binary maps of the simulated pressure field that falls below the nonlinear threshold of each acoustic reporter, and above the collapse pressure of each acoustic reporter. (**d**) Unmixing results of the simulated bARG_560_ (*top row*) and bARG_710_ (*bottom row*) images using the depth-correcting multiplexing matrix, *M*_2d_.

We anticipate several improvements and extensions to this technology. One limitation of acoustic pressure-based multiplexing is that it requires the ability to apply controlled pressure levels within tissues. Although we had no difficulty doing so in mouse tumors and colons, it may be more challenging in more attenuating scenarios. To address such cases, it may be necessary to develop pulse sequences that estimate attenuation aberration and correct for it by shaping transmitted pulses. Alternatively, for simple cases where attenuation is uniform, depth-dependent functions for each acoustic reporter can be created for effective unmixing with an attenuation-correcting multiplexing matrix, *M*_2d_ (Extended Data Fig. 9).

Beyond microbial applications, an important extension of the technology would be to implement acoustic multiplexing in mammalian ARGs, which are being used to image a variety of cell types in vivo, including tumors and immune cells. The presence of *gvpC* in mammalian ARGs suggests that an approach analogous to the one taken here could uncover pressure-shifted variants, although they will probably need to be screened in the mammalian context.

Another valuable extension would be to add more ARG ‘sounds’ to enable higher-order multiplexing. Just as fluorescent proteins have been mined genomically, evolved and engineered for decades to generate an array of reporter genes for multiplexed imaging, which have since been used in innumerable optical imaging applications, the methods and constructs developed here suggest that a similar trajectory may be available for ultrasound.

## Methods

### Molecular biology

For all genetic constructs, primers were designed manually and ordered from Integrated DNA Technologies, and all enzymes were ordered from New England Biolabs (NEB) unless otherwise stated. The constitutive promoter used in our circuits was a gift from H. Shuman (Addgene plasmid no. 84821). Polymerase chain reactions (PCRs) were amplified using *Taq* (cat. no. M0270, NEB) for fragments <3 kb and Q5 (cat. no. M0492, NEB) for fragments >3 kb, and Gibson assembly was carried out using the HiFi DNA Assembly Master Mix (cat. no. E2621, NEB). All assembly reactions were transformed into Stable *E. coli* (cat. no. C3040, NEB), except for *pBAD* promoter mutagenesis where transformations were performed directly on attenuated Stm. All constructs were verified via sequencing.

### Manufacturing dual-layer 96-well agar plates

Lennox Luria broth (LB) agar was prepared, autoclaved and allowed to cool to 65 °C before any reagents were added to it. Wells were first loaded with 125 µl of agar containing the relevant induction reagents (for example, IPTG, l-arabinose) and antibiotics, and incubated at room temperature for ~10 min to allow this first layer to polymerize. The suppression layer was then loaded into each well as a second 125-µl volume, this time containing the relevant suppression reagents (for example, d-glucose) and antibiotics. The second layer was allowed to polymerize and cool to ≤37 °C before bacteria patching. Plates were prepared within 2 h of bacteria patching.

### Cell culture and cell line generation

BL21-AI *E.* *coli* was acquired from Invitrogen (cat. no. C607003), attenuated Stm was a gift from J. Hasty (University of California, San Diego), and EcN was acquired from Mutaflor. Unless otherwise stated, all plasmids were introduced into bacteria via electroporation, followed immediately by addition of SOC medium containing 0.5% d-glucose (m/v) and incubation for 2–3 h at 250 rpm and 37 °C before introduction of any antibiotics. Cells were then inoculated in Lennox LB containing 0.5% d-glucose (m/v) with antibiotics and grown at 250 rpm, 37 °C for 6–8 h to reach log phase ahead of patching on 96-well dual-layer agar plates, or on 10-cm dual-layer agar plates for mass harvesting.

### Cell patching for solid-phase expression

For patching, 5–8 µl of bacteria were dispensed onto agar, and incubated at 37 °C for 24 h to allow for gene expression. The *gfp* placed within the GV operon of bARG_560_ was used as a troubleshooting control during assembly screening (Fig. 1b). Operon expression (with a readout of GFP fluorescence) did not necessarily result in GV assembly, but served to let us know that the operon was indeed being induced for expression. Bacteria exhibited visible increases in patch opacity in cases of successful GV assembly. In more descriptive terms, bacteria patches appeared strikingly white if they contained GVs, relative to negative control patches or patches that failed to successfully assemble GVs that appeared yellow-gray (Fig. 1b and Extended Data Fig. 5). Cells were then collected by dispensing 100 µl of PBS and incubating at room temperature for ~10 min, before mixing the sample and collecting it into a secondary apparatus for downstream measurements. Bacteria exhibiting successful GV assembly should start to float in PBS within the 10-min incubation period. GV assembly was induced on solid-phase agar in 96-well format unless otherwise stated.

### GvpC library construction and expression

All PCR reactions were performed using the Taq Hot Start System (cat. no. M0496, NEB) and fragment insertion was conducted with the High Fidelity DNA Assembly System (cat. no. M0541, NEB). Random mutagenesis was conducted using error-prone PCR of the complete *gvpC* sequence (UniProt P09413) by adding 20–250 μM MnCl_2_ to the reaction and amplifying across 30 cycles. Because the *gvpC* sequence includes repeat regions, we were concerned that low mutation rates may not result in substantially different acoustic properties from that of the wild type. On the opposite end, a high mutation rate may result in GvpC proteins that fail to adhere to the GV shell entirely. We therefore sought to create a GvpC library with sizable variation in its mutation rate, and accomplished this by applying a large range of MnCl_2_ concentrations, and then pooled all reactions to form a single library. Our mean mutation rate was 5.36 mutations with an s.d. of 13.5 mutations. PCR products were then cloned into the ΔgvpC plasmid backbone, replacing *gfp*, and electroporated into BL21-AI *E.* *coli* (cat. no. C607003, Invitrogen). The pooled bacteria population was spread onto 10-cm dual-layer agar plates containing 60 µM IPTG, 0.3% l-arabinose (m/v), 0.5% d-glucose (m/v) and 2× kanamycin, and incubated overnight at 37 °C. Single white colonies were picked to inoculate 1.5-ml volumes of Lennox LB containing 2% d-glucose and 2× kanamycin and grown at 250 rpm, 37 °C for 6–8 h, before being patched onto 96-well dual-layer agar plates containing 60 µM IPTG, 0.3% l-arabinose (m/v), 0.5% d-glucose (m/v) and 2× kanamycin, and incubated at 37 °C for 24 h for expression.

### Ultrasound imaging

All agarose solutions used for ultrasound imaging were passively degassed for at least 24 h before casting. Cells suspended in PBS were mixed 1:1 (v/v) with 1% low-melt agarose (m/v) and loaded into imaging phantoms made of 2% agarose (m/v). Imaging was performed using a Verasonics Vantage programmable ultrasound scanning system and a L22-14vX 128-element linear array Verasonics transducer, with a specified pitch of 0.1 mm, an elevation focus of 8 mm, an elevation aperture of 1.5 mm and a center frequency of 18.5 MHz with a 67% −6 dB bandwidth. All image acquisition scripts were coded on MATLAB (Mathworks). In vitro samples were centered at a depth of 5 mm during acquisition. For nonlinear imaging, a custom cross-wave (X-wave) amplitude modulation (xAM) sequence with a cross angle (*θ*) of 19.5°, an aperture of 65 elements and a transmit frequency of 15.625 MHz was used. Each image was an average of 30 accumulations. For linear image acquisition, a conventional ray-line scanning B-mode pulse sequence with parabolic focusing at 10 mm and an aperture of 40 elements was used at the minimum transmit voltage (1.6 V). A parabolic B-mode pulse set at the maximum transmit voltage (30 V) was used to collapse GVs. The transmitted pressure at the sample position at 5 mm was measured using a Precision Acoustics fiberoptic hydrophone system.

### Automated acoustic screening

Data collection for the library included acquisition of nonlinear images at increasing peak positive pressures (330–1,840 kPa), followed by a parabolic B-mode pulse and a final postcollapse nonlinear image at the maximum peak positive pressure (‘postcollapse nonlinear image’, 1,840 kPa). Linear imaging was also performed on each sample (‘B-mode’, 350 kPa). These additional measurements were implemented to (1) automate region-of-interest segmentation (B-mode), (2) filter out samples that contained very low GV concentrations and therefore unreliable nonlinear signals (B-mode) and (3) exclude samples that were confounded by artifacts in their FOVs (postcollapse nonlinear image). In more detail:

∘ B-mode images were used to automate region-of-interest placement for each sample, as B-mode images included a reliable, high contrast edge at the top of each sample. This region-of-interest was then overlaid on the nonlinear image series of each sample to output the mean nonlinear signal of each mutant as a function of peak positive pressure.
∘ The signal intensity of each sample on B-mode served as a proxy for the concentration of GVs in each sample, independent of nonlinear behavior (Extended Data Fig. 1). Most samples produced enough GVs for reliable nonlinear signal measurements. On rare occasions, however, some samples had low GV production, resulting in low nonlinear signal yields. To exclude these samples from our dataset, we applied a B-mode filter to our pipeline. In other words, we were assured that low nonlinear signals on our acoustic map (Fig. 1b) was indeed due to the low nonlinearity of those individual mutant GVs, and not due to poor GV production of that sample.
∘ On other rare occasions, artifacts appeared within FOVs of samples, for example from reflection from bubbles that formed during sample loading that generated nonlinear signals. Since readouts of samples confounded by artifacts are not reflective of the ‘true’ nonlinear signals of that GV mutant, a sample with high nonlinear signals in its postcollapse nonlinear image was excluded from the library.

After filtering our library data, all remaining mutants were characterized by two parameters of their nonlinear signal pressure functions:

∘ The nonlinear signal threshold (*P*_o_) was defined as the peak positive pressure at which nonlinear signals were first ‘observed’, that is, statistically greater than nonlinear signals of bacteria expressing GFP measured at the same pressure (Fig. 1d).
∘ The maximum nonlinear yield (*S*_max_) was defined as the maximum nonlinear signal that was yielded within the ‘nondestructive pressure window’, which has an upper limit defined by the collapse pressure of bARG_560_ (1,220 kPa).

### Linear unmixing of acoustic signals

All image processing scripts were coded on MATLAB (MathWorks). To generate the multiplexing matrix ‘*M*’, reference samples of Stm encoding bARG_560_ and EcN encoding bARG_710_ were imaged using the nonlinear pulse sequence across the pressure domain. During experiments, image acquisition initially included application of pressures above the collapse pressure of bARG_560_ due to uncertainties in the voltage-to-pressure conversion, but these images were removed from the tail-end of the image series upon pressure calculation, before unmixing. Regions of interest were segmented for each sample and organized into an *N* × *P* matrix, where *N* is the number of acoustic reporters, *P* is the number of pressure acquisitions and the value in each cell represents the nonlinear signal for a given reporter at a given pressure. Once *M* was established, all unknown images thereafter were acquired following the same acquisition parameters as the reference samples. Unknown images were first thresholded (~100 a.u. for in vitro samples, ~200 a.u. for in vivo images), such that if the nonlinear signal of a pixel did not surpass this threshold at any pressure acquired, it was zeroed out on the image and, by extension, on the coefficient matrix ‘*C*’. Linear unmixing was then performed pixelwise to solve for the coefficient matrix ‘*C*’ to generate a matrix of size *Z* × *X* × *N*, where *Z* is the depth and *X* is the width of the deconvoluted image. The following equation was used to solve for *C*, using a non-negative least squares fitting, where *λ* is the nonlinear signal as a function of pressure for each pixel:

$${C}=\frac{\lambda }{{M}}$$

The coefficient matrix ‘*C*’ contains two planes in the third dimension that represent the relative contributions of each acoustic reporter to the raw signal being measured, which we call our ‘acoustic channel’ images. For all samples, the acoustic channel images of each sample are scaled to the same upper limit. Different samples are not necessarily scaled to the same upper limit due to variability in the concentration of GV-expressing bacteria between samples subjected to different experimental conditions. Meanwhile, a lower limit of zero is applied to all acoustic channel images. We calculated a normalized difference (ND) between the acoustic channel images for each sample, where *S*_560_ is the signal from the 560 acoustic channel and *S*_710_ is the signal from the 710 acoustic channel (Extended Data Fig. 6):

$${\rm{ND}}=\frac{{S}_{560}-{S}_{710}}{\sqrt{({S}_{560}^{2}-{S}_{710}^{2})}}$$

### Mammalian cell culture

Murine colon carcinoma cells (MC26) were acquired (cat. no. 400156, BioHippo) and grown in Dulbecco’s modified Eagle’s medium supplemented with 10% fetal bovine serum (FBS) and 1× antibiotic-antimycotic.

### Animal models

All animal protocols were approved by the Institutional Animal Care and Use Committee at the California Institute of Technology (Protocol no. 1735) and comply with federal and state regulations governing the humane care and use of laboratory animals. Animals were housed in a facility maintained at 21–24 °C and 30–70% humidity, with a lighting cycle of 13 h on (06:00–19:00) and 11 h off. Female BALB/c mice (Strain no. 000651, Jackson Laboratory) were used for all animal experiments. Mice were anesthetized with 1–3% isoflurane in 100% oxygen using a nose cone colinked to a vacuum line for active scavenging, and kept warm on a heated stage.

### Ultrasound imaging of the GI tract

For imaging the GI tract, mice were placed in the supine position, depilated over the imaging region and received an enema with PBS to clear the tract of feces using a flexible plastic tubing needle tip (cat. no. CAD9918, Millipore Sigma). Before imaging, Stm encoding bARG_560_ and EcN encoding bARG_710_ were induced for GV expression as described above, collected and diluted in pure or mixed populations (1:1 v/v) to a concentration of 2 × 10^8 ^cells ml^−1^, and then mixed in a 1:1 ratio with 42 °C 1% low-melt agarose. An eight-gauge needle was filled with the gelled bacteria solution and incubated at room temperature to polymerize. The cooled bacteria-embedded gel was then injected into the colon of the mouse with a PBS back-filled syringe. B-mode imaging was used to identify the colon lumen. Nonlinear ultrasound imaging was then performed as described above.

To ensure that the nonlinear signals we were observing arose from GVs and not from the interface of the GI tract with its lumen and/or extraluminal fluid of the peritoneal cavity, we also applied a parabolic B-mode collapse pulse to irreversibly collapse the GVs and acquired postcollapse nonlinear images (Extended Data Fig. 8). Following acquisition, we processed the raw image series by filtering out pixels that did not generate significant nonlinear signals at any point throughout the acquisition series (*S* < 200 a.u.). In some cases, we also discarded single pressure acquisitions from the image series because of non-negligible movement from the mouse during breathing. For analysis, we segmented regions of interest representing the GI lumen from the raw nonlinear image, which was then overlaid on the coefficient matrices to quantify mean signal values from each acoustic channel.

### In vivo ARG expression by tumor-colonizing bacteria

Mice were injected subcutaneously at the right flank with 3 × 10^5^ MC26 cells and solid tumors formed over 2–3 weeks (200–400 mm^3^). The day before injection of bacteria, ibuprofen was added to the drinking water at 0.2 mg ml^−1^. Stm encoding bARG_560_ and EcN encoding bARG_710_ were inoculated in Lennox LB containing 0.5% d-glucose (m/v) with antibiotics and grown at 250 rpm, 30 °C for 12–16 h. Cells were then washed three times with PBS and diluted to OD_500_ = 0.300; 150 µl of single- or dual-species bacteria solutions were then injected intravenously into the tail veins of mice. Cells were given 24 h following intravenous injection to colonize tumors, after which mice were injected intraperitoneally every 24 h for 3 days with 120 mg of l-arabinose to induce expression of bARG_560_ and/or bARG_710_. On the fourth day, mice were placed in a prone position, depilated over the imaging region and nonlinear ultrasound imaging was performed on a cross-section of the tumor. Mice were euthanized immediately after ultrasound imaging for histology.

### Ultrasound simulations

The impacts of noise and depth on acoustic multiplexing were investigated using simulations performed on MATLAB (MathWorks). For depth simulations, we applied a biologically relevant attenuation rate of 0.5 dB cm^−1 ^MHz^−1^ at a frequency of 15.625 MHz and a range of source amplitudes to simulate a series of pressure fields that matched experimental hydrophone measurements at a depth of 5 mm^37^ (Extended Data Fig. 9a). We then used the simulated pressure field and the pressure-signal spectra of each reporter to generate simulated nonlinear signal images of our reporters across the entire field of view, including collapse effects at pressures greater than the collapse threshold (Extended Data Fig. 9b,c). The reporters were modeled to be uniformly distributed within the field of view. Because the collapse pressure threshold of bARG_560_ begins to be exceeded in the near-field at higher source amplitude pressures (>1,240 kPa), we limited the pressure window to an upper limit of 1,200 kPa. Finally, to examine one potential approach for attenuation correction, we performed depth-corrected linear unmixing using a multiplexing matrix we are calling *M*_2d_ (Extended Data Fig. 9d). In simple terms, *M*_2d_ generates a unique multiplexing matrix *M*(*d*) for linear unmixing at depth, *d*, to deconvolve acoustic reporter contributions based on both the signal spectra of that pixel and its depth.

To investigate the effects of noise on acoustic multiplexing, we applied random noise to raw images of tumors at increasing levels of intensity, according to the following equation:

$$I(m)={I}_{0}+{mR}$$

where ${I}_{0}$ is the original image series, $m$ is a user-controlled multiplier and $R$ is a matrix of random values that is equal in size to ${I}_{0}$ with a mean of 0 and an s.d. that matches that of background tissue at each pressure acquisition in the original raw image series ${I}_{0}$. We assessed the degradation of contrast-to-noise ratios (CNR) of the raw nonlinear images at increasing levels of added noise, using the following formula, where *S*_tumor_ is the mean signal intensity of the tumor region, *S*_background_ is the mean signal intensity of background tissue and *σ*_background_ is the s.d. of the signal intensity of background tissue:

$${\rm{CNR}}=\frac{|{S}_{{\rm{tumor}}}-{S}_{{\rm{background}}}|}{{\sigma }_{{\rm{background}}}}$$

**Extended Data Fig. 10:**
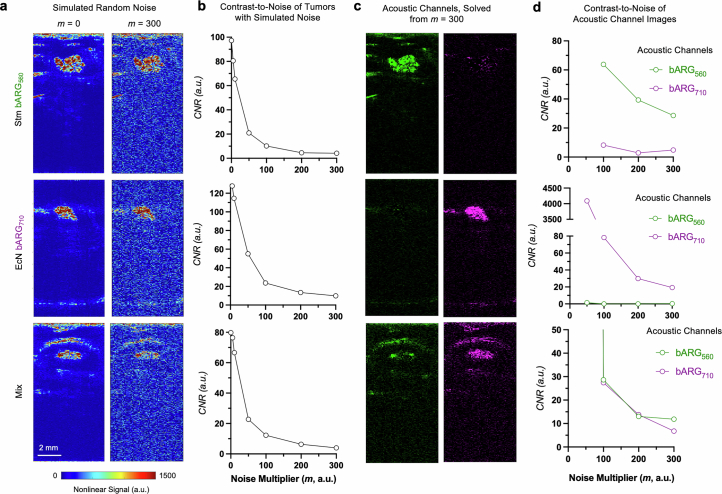
Effects of Noise on Deconvolution. (**a**) Representative nonlinear ultrasound images of tumors of mice that were injected with attenuated *S. typhimurium SL1344* (Stm) encoding bARG_560_ and/or *E. coli Nissle 1917* (EcN) encoding bARG_710_ (noise multiplier, *m*=0), and with added noise (*m*=300). Scale bar, 2 mm. (**b**) Contrast-to-noise ratio (CNR; a.u.) of tumor images with variable levels of added noise. (**c**) Acoustic channel images that were deconvoluted from tumor images where *m*=300. (**d**) Contrast-to-noise ratio (CNR; a.u.) of acoustic channel images resulting from deconvolution of tumor images with variable levels of added noise. Source data

We performed linear unmixing, as previously described, on these new image series, and then assessed deconvolution performance by calculating the CNR of the acoustic channel images at increasing levels of added noise (Extended Data Fig. 10).

### Histology

Whole-tumors were excised from mice immediately after ultrasound imaging and placed in 4% paraformaldehyde for at least 48 h. Following fixation, tumors were moved into 30% sucrose (m/v) for at least 24 h, or until they sank to the bottom of the solution. Tumors were dried for approximately 30 min with paper towel, positioned in a volume of OCT freezing medium and incubated at −30 °C overnight. Tumors were then cut into 20-µm-thick sections and placed onto microscope slides using a Leica CM1950 cryostat. Fluoro-gel Mounting Medium (cat. no. 17985-50, Electron Microscopy Sciences) was applied dropwise to tumor sections, and then encased with glass coverslips. Slides were imaged with a Zeiss LSM 800 Confocal Laser Scanning Microscope with a ×5 objective for whole-tumor tiled imaging, and ×20 objective for localized imaging of the tumor core and tumor rim.

### Statistical analysis

No statistical methods were used to predetermine sample size. All *n* values represent biological replicates. Error bars indicate s.e.m., unless otherwise stated. A threshold of 0.05 was used for statistical significance. *P* values were calculated using a two-tailed paired *t*-test, an ordinary one-way analysis of variance (ANOVA), or a two-way ANOVA.

### Reporting summary

Further information on research design is available in the Nature Portfolio Reporting Summary linked to this article.

## Online content

Any methods, additional references, Nature Portfolio reporting summaries, source data, extended data, supplementary information, acknowledgements, peer review information; details of author contributions and competing interests; and statements of data and code availability are available at 10.1038/s41592-025-02825-w.

## Supplementary information

Supplementary Information Supplementary Notes 1–2 and Tables 1–3.

Reporting Summary

## Source data

Source Data Fig. 1 Statistical source data.

Source Data Fig. 2 Statistical source data.

Source Data Fig. 3 Statistical source data.

Source Data Fig. 4 Statistical source data.

Source Data Fig. 5 Statistical source data.

Source Data Extended Data Fig. 1 Statistical source data.

Source Data Extended Data Fig. 2 Statistical source data.

Source Data Extended Data Fig. 4 Statistical source data.

Source Data Extended Data Fig. 5 Statistical source data.

Source Data Extended Data Fig. 6 Statistical source data.

Source Data Extended Data Fig. 10 Statistical source data.

## Data Availability

The main plasmids used in the study will be available through Addgene. All other constructs can be made available by the corresponding author under a material agreement with the California Institute of Technology. Raw data can be made available by the corresponding author upon reasonable request. Source data are provided with this paper.

## Code availability

Processing scripts used to generate key figures and results will be posted to https://github.com/shapiro-lab.
