# Supplementary material for: Multiplexed ultrasound imaging of gene expression
Source: Nat Methods. 2025 Nov 18;22(12):2594–600. doi: 10.1038/s41592-025-02825-w (PMC12695634; doi:10.1038/s41592-025-02825-w)
Supplement: Supplementary file 1 — Supplementary Notes 1–2 and Tables 1–3. [file 41592_2025_2825_MOESM1_ESM.pdf]

nature methods

Supplementary information https://doi.org/10.1038/s41592-025-02825-w

Multiplexed ultrasound imaging of gene expression

In the format provided by the authors and unedited

TABLE OF CONTENTS

Supplementary Note 1. Structural and mechanistic predictions of $GvpC_{L154P}$

Supplementary Note 2. Achieving $bARG_{560}$ and $bARG_{710}$ expression in probiotic strains

Supplementary Table T1. Circuit variables and parameter limits for GV expression by $bARG_{560}$.

Supplementary Table T2. Acoustic properties and protein sequences of sampled mutants from the GvpC Mutagenesis Library.

Supplementary Table T3. The Multiplexing Matrix, *M*, for linear unmixing of acoustic reporter signals.

Supplementary Note 1 - Structural and mechanistic predictions of $GvpC_{L154P}$

Because of the restricted rotation of its N-C_α bond, and lack of hydrogen atom for hydrogen bonding, we expected the proline to disrupt the alpha helical structure of GvpC in some way. We processed the mutated sequence using AlphaFold to generate a structural prediction with a confidence score (pLDDT) of 76.9%. First, we found that L154 was part of the shell-binding face of GvpC, resulting in direct loss of a single shell-binding residue. Additionally, L154P generated a significant structural kink that rigidly deviates the final 21% length of the GvpC helix away from its binding interface on the shell ($\Delta\alpha$ = 12.1° relative to wild-type; **Extended Data Fig. 3**)

For example, the next shell-binding residue after L154P, F161, was situated 2.1 Å from its original alignment, which is enough to weaken, if not disrupt, its speculated interaction with E60 of gvpA1 (**Extended Data Fig. 3**). Binding residues further downstream were increasingly misaligned. These measurements were made on the free-form structure of GvpC, which may differ from analysis performed on a shell-bound structural analysis of GvpC, but because knowledge of the binding mechanisms between the GvpA shell protein and the GvpC scaffolding protein is still limited, it is unlikely that this type of analysis will yield any more tangible insight at this point.

Supplementary Note 2 - Achieving bARG$_{560}$ and bARG$_{710}$ expression in probiotic strains

We transitioned our constructs from T7-dependent circuits to the directly inducible *pBAD* promoter, but initially observed no GV assembly from either construct (bARG$_{560}$, bARG$_{710}$) in either strain (Stm, EcN) across a range of induction conditions. First, we reasoned that removal of genes not directly contributing to GV assembly could reduce burden and increase GV yield through reallocation of finite transcriptional and translational resources.

We tested single gene deletions of *gvpW*, *gvpX*, *gvpY*, *gvpH*, and *gvpZ* in BL21-AI *E. coli*, as these were already deemed nonessential for GV assembly in the native organism, in addition to deletions of *gvrB* and *gvrC*, as these were putative regulatory proteins that would be obsoleted by our synthetic control circuit. We saw GV yield increase across all of our single gene deletion constructs relative to the original wild-type construct (bARG$_{ser}$), in agreement with our finite resources hypothesis (**Extended Data Fig. 4**).

We then tested a stepwise gene deletion tree in EcN, starting with bARG$_{560}$ as the parent construct (*gfp*$^{+}$, Δ*gvpC*), and found that combined deletions of *gvpW*, *gvrB* and *gvrC* enabled GV assembly in this microbe across a range of induction conditions (**Extended Data Fig. 5**). Next, we moved this new, smaller bARG$_{560}$ construct into Stm, but again observed no assembly. We then targeted the I1 and I2 operators of *pBAD* for mutagenesis, and found a mutant that enabled GV assembly from bARG$_{560}$ in Stm (**Extended Data Fig. 5**).

Supplementary Table T1. Circuit variables and parameter limits for GV expression by bARG$_{560}$.

Variable		Parameter Limits for GV Assembly
1	bARG Operon T7 RNA Polymerase-dependent Promoter Circuit	pLac
2	bARG Operon Inducer Concentration	IPTG, 40–70 μM
3	T7 RNA Polymerase Operon Suppressor Concentration	D-glucose, ≤0.5% (m/v)
4	T7 RNA Polymerase Operon Promoter Circuit (Expression Strain Background)	pBAD (BL21-AI™, cat no. C607003)
5	T7 RNA Polymerase Operon Inducer Concentration	L-arabinose, 0.3–0.5% (m/v)
6	NaCl Concentration	5–10 g/L
7	Induction Temperature	30–37°C

Supplementary Table T2. Acoustic properties and protein sequences of sampled mutants from the GvpC Mutagenesis Library.

Po	maxSignal	Protein Sequence
749.4295	2024.64151	MISLMAKIRQEHQSIAEKVAELSLETREFLSVTTAKRQEQAEKQAQELQAFYKDLQETSQQFLSETAQARIAKLKNKLKNC*
787.169	1774.33682	MISLMAKIRQEHQSIAEKVAELSLETREFLSVTTAKRQEQAEKQAQELLAFYQEVRETSQQFLSETAQARIAQAEKQAQELLAFHKELQETSQQFLSATADARTAQAKEQKESLLKFRQDLFVSIFG*
862.648	1637.36536	MISLMAKIRQEHQSIAEKVAELSLETREFLSVTTAKRQEQAEKQAQELQAFYKDLQETSQQFLSETAQARIAQAEKQAQELLAFHKELQEXXSAVFISNSRRKXLLKPKETEXNLXXNFXXGFVXXVSLG*
862.648	1646.55514	MISLMAKIRQEHQSIAEKVAELSLETREFLSVTTAKRQEQAEKQAQELQAFYKDLQETSQQFLSETAQARIAQAEKQAQELLAFHKXLXXXSQQFLSATAPSQNCSS*
900.3875	1434.84752	MISLMAKIRQEHQSIAEKVAELSLETREFLSVTTAKRQEQAEKQAQELQAFYKDLQETSQQFLSETAQARIAQAEKQAQELLAFHKELQETSQQFLSATAQXXIXXXEXKRKNC*
900.3875	1876.79095	MISLMAKIRQEHQSIAEKVAELSLETREFLSVTTAKRQEQAEKQAQELLAFHKELQETSQQFLSATADARTAQAKEQKESLLKFRQDLFVSIFG*
862.648	1163.02145	MISLMAKIRQEHQSIAEKVAELSLETREFLSVTTAKRQEQAEKQAQELQAFYKDLQETSQQFLSETAQARIAQAEKQAQELLAFHKELQETSQQFLSATAPRPEFAQS*
787.169	1099.28627	MISLMAKIRQEHQSIAEKVAELSLETREFLSVTTAKRQEQAEKQAQELQAFYKDLQETSQQFLSETADARTAQAKEQKESLLKFRQDLFVSIFG*
824.9085	1049.9068	MISLMAKIRQEHQSIAEKVAELSLETREFLSVTTAKRQEQAEKQAQELQAFYKELQETSQQFLSETAQARIAQAEKQAQELLAFHKELQETSQQFLSATADARTAQAKEQKESLLKIPSGFVCEYLWLXSFXRRLAXKVSGSPSAXKDPFGTWAAKXSXST*
787.169	1042.17713	MISLMAKIRQEHQSIAEKVAELSLETREFLSVTTAKRQEQAEKQAQELLAFHKELQETSQQFLSATADARXAXAXNRXNLSXXPSXFXXXXXX*
749.4295	1397.46955	MISLMAKIRQEHQSIAEKVAELSLETREFLSVTTAKRQEQAEKQAQELQAFYKDLQETSQQFLSETAQARIAQAEKQAQELLAFHKELQETXQQFLSATAQA*
862.648	1188.23953	MISLMAKIRQEHQSIAEKVAELSLETREFLSVTTAKRQEQAEKQAQELQAFYKDLQETSQXFLSETAQARIAQAEKQAQELLAFHKELQETSQQFLSATADARTAQAKEQKESLLKFRQDLFVSIFG*
787.169	1843.27645	MISLMAKIRQEHQSIAEKVAELSLETREFLSVTTAKRQEQAEKQAQELQAFXKELQETSQQFLSETAHARIAHAEKQAQELLSFHKELHETSQNCLSATAQARIAQAEKQTQELLAFYQEVRETRQHCLS*
787.169	1752.61045	MISLMAKIRQEHQSIAEKVAELSLETREFLSVTTAKRQEQAEKQAQELLAFHKELQETSQQFLSATADARTAXAKEQKESLLKFRQDLFVSIFGXTSFVGAW
824.9085	1365.28312	MISLMAKIRQEHQSIAEKVAELSLETREFLSVTTAKRQEQAEKQAQELQAFYKDLQETSQQFLSETAQARIAQAEKQAQELLAFHXELQETSQQFLSATADARTAQAKEXXESLLKFRXDLFMXXFGXX
787.169	2287.67572	MISLMAKIRQEHQSIAEKVAELSLETXEFLSVTTAXR
824.9085	1444.34284	MISLMAKIRQEHQSIAEKVAELSLGTREFLSVTTAKRQEQAEEQAQEPLAFHKELQETSQQFLSXTADARTAQAKEQKESLLKFRQDLFVSIFG*
862.648	2053.72393	MISLMAKIRQEHQSIAEKVAELSLETREFLSVTTAKDKSKLKNKLKNC*
824.9085	1456.22801	MISLMAKIRQEHQSIAEKVAELSLETREFLSVTTAKRQEQAEKQAQELQAFYKDLQETSQQFLPETAQARIAQAEKQAQELLAFHKELQETSQQFLSATADARTAQAKEQKESLLKFRQDLFVSIFG*

824.9085	1446.48497	MISLMAKIRXEHQSIAEKVAELSLETREFLSVTTAKRQXQAEXQAQELQAFYKDLQETXXX
824.9085	2078.43239	MISLMAKIRQEHQSIAEKVAELSLETREFLSVTTAKRQEQAEKQAQELQAFYKDLQETSQQFLSETAQARIAQAEKQAQELLAFHKELQETSQQFLSATADARTAQAKEQKEPLLKFRQDLFVSIFG*
787.169	2378.5067	MISLMAKIRQEHQSIAEKVAELSLETKEFLSVTTAKRQEQAEKQAQELQAFYKDLQETSQQFLSATADARTAQAKEQKESLLKFRQDWYEIRFG*
900.3875	1515.1613	MISLMAKIRQEHQSIAEKVAELSLETREFLSVTTAKRQEQAEKQAQELLAFYKELQETSQQFLSETAQARIAQAEKQAQELLAFHKELQETSQQFLSATAQARIAQAKEQKESLLKFRQDLFVSIFG*
900.3875	854.073207	MISLMAKIRQEHQSIAEKVAELSLETREFLSVTTAKRQEQAEKQAQELQAFYKDLQETSQQFLSATAHARTAQAKEQKESXLKFRXDLFVSIFG*
862.648	1073.62673	MISLMAKIRQEHQSIAEKVAELSLGTREFLSVTTAKRQEQAEKQAQELLAFHKELQETSQQFLSATADARTAQAKEQKESLLKFRQDLFVSIFG*
1126.825	546.52509	MISLMAKIRQEHQSIAEKVAELSLETREFLSVTTAKRQEQAEKQAQELLAFYQEVRETSQQFLSATAQARIAQAEKQAQELLAFHKELQETSQQFLSATAQARIAQAEKQAQELLAFYQEVRETSQQFLSATAQARIAQAEKQAQELLAFHKELQETSQQFLSTTADARTAQAKEQKESLLKFRQDLFVSIFG*
1013.606	696.166918	MISLMAKIRQEHQSIAEKVAELSLETREFLSVTTAKRQEQAEKQAQELLAFYQEVRETSQQFLSATAQARIAQAEKQAQELLAFHKELQETSQQFLSATAQARIAQAEKQAQELLAFYQEVRETSQQFLSATAQARIAQAEKQAQELLAFHKELQETSQQFLSXTADARTAQAKEQKESLLKFRQDLFVSIFG*
1051.346	697.85816	MISLMAKIRQEHQSIAEKVAELSLETREFLSVTTAKRQEQAEKQAQELQAFYKDLQETSQQFLSETAQARIAQAEKQAQELLAFHKELQETSQQFLSATAQARIAQAERQAQELLAFYQEVRETSQQFLSATAQARIAQAEKQAQELLAFHKELQETSQQFLSATADARTAQAKEQKESLLKFRQDLFVISLVKLVCRRLAVKVR*
975.8665	1079.40827	MISLMAKIRQEHQSIAEKVAELSLETREFLSVTTAKRQEQAEKQAQELQAFYKDLQETSQQFLSETAQARIAQAEKQAQELLAFYQEVRETSQQFLSATAQARIAQAEKQAQELLAFHKELQETSQQFLSATADARTAQAKEQKESLLKFRQDLFVSIFG*
1051.346	1051.3455	MISLMAKIRQEHQSIAEKVAELSLETREFLSVTTAKRQEQAEKQAQELQAFYKDLQETSQQFLSETAQARIAQAEKQAQELLAFHKELQETSQQFLSATAQARIAQAEKQAQELLAFYQEVRETSQQFLSATAQARIAQAEKQAQELLAFHKELQETSQQFLSATADARTAQAKEQKESLLKFRQDLFVSGEHQTYRDYFQIGALELERWRRTREREAASSRIASIDERIADIDKEKAALLADATAASAVAENNDKSXXXXXXEKNXXWXXKXXXWRLSLXXKXXXPSXXYPXXRXXXXX
975.8665	798.382499	MISLMAKIRQEHQSIAEKVAELSLETREFLSVTTAKRQEQAEKQAQELQAFYKDLQETSQQFLSETAQARIAQAEEQAQELLASHKELQETSQQFLSATAQARIAQAEKQAQELLAFYQEVRETSQQFLSATAQARIAQAEKQAQELLAFHKELQETSQQFLSATADARTAQAKEQKESLPKFRQDLFVSIFG*
1051.346	676.947998	MISLMAKIRQEHQSIAEKVAELSLETSEFLSVTTAKRQEQAEKQAQELQAFYKDLQETSQQFLSETAQARIAQAEKQAQELLAFHKELQETSQQFLSATAQARIAQAEKQAQELLAFYQEVRETSQQFLSATAQARIA*
975.8665	586.626474	MISLMAKIRQEHQSIAEKVAELSLETREFLSVTTAKRQEQAEKQAQELQAFYKDLQETSQQFLSETAQARIAQAEKQAQELLAFHKELQETSQQFLSATAKPELLKLKNKRKNC*
1126.825	671.339733	MISLMAKIRQEHQSIAEKVAELSLETREFLSVTTAKRQEQAEKQAQELQAFYKDLQETSQQFLSETAQARIAQAEKQAQELLAFHKELQETSQQFLSATAQARTAQAEKQAQELLAFYQEVRETSQQFLSATAQARIAQAEKQAQELLAFHKELQETSQQFLSATADARTAQAKEQKESLLKFRQDLFVSIFG*
1126.825	508.16395	MISLMAKIRQEHQSIAEKVAELSLETREFLSVTTAKRQEQAEKQAQELQAFYKDLQETSQQFLSATAQARIAQAEKQAQELLAFHKELQETSQQFLSATADARTAQAKEQKEPLLKFRQDLFVSIFG*

1089.085	667.591339	MISLMAKIRQEHQSIAEKVAELSLETREFLSVTTAKRQEQAEKQAQELQAFYKDLQETSQQFLSATAQARIAQAEKQAQELLAFHKELQETSQQFLSATADARTAQAKEQKESLLKFRQDLFVSIFG*
1089.085	688.222328	MISLMAKIRQEHQSIAEKVAELSLETREFLSVTTAKRQEQAEKQAQELQAFYKDLQETSQQFLSETAQARIAQAEKQAQELLAFHKELQXTSQQFLSATAQARIAQAEKQAQELLAFYQEVRETSQQFLSATAQARIAQAERQAQELQAFYKDLQETSQQFLSETAQARIAQAEKQAQELLAFHKELQETSQQFLSATAQARIAQAEKQAQELLAFYQEVRETSQQFLSATAQARIAQAEKQAQELLAFHKGTXRNKSAVFISSSPXQELLKXKEQXXSLLKFRXDLFXXXXFXXXXFXXX
1051.346	588.51129	MISLMAKIRQEHQSIAEKVAELSLETREFLSVTTAKRQEQAEKQAQELQAFYKDLQETSQQFLSATAQARIAQAEEQAQELLAFHKELQETSQQFLSATADARTAQAKEQKESLLKFRQDLFVSIFG*
1013.606	620.742706	MISLMAKIRQEHRSIAEKVAELSLETREFLSVTTAKRQEQAEKQAQELQAFYKDLQETSQQFLSETAQARIAQAEKQAQELLAFHKELQETSQQFLSATAQARIAQAEKQAQELLAFYQEVRETSQQFLSATAQARIAQAEKQAQELLAFHKELQETSQQFLSATADARTAQAKELKESLLKFRQDLFVSIFG*
1051.346	586.621704	MISLMAKIRQEHQSIAEKVAELSLETREFLSVTTAKRQEQAEKQAQELQAFYKDLQETSQQFLSETAQARIAQAEKQAQELLAFHKELQETSQQFLSATAQARIAQAEKQAQELLAFYQEVRETSQQFLSATAQARIAQAEKQAQELLAYHKELQETSQQFLSATADARTAQAKEQKESLXKFRQDLFVSIFG*
975.8665	1548.06548	MISLMAKIRQDHQSIAEKVAELSLETRGFLSVTTAKRQEQAEKQAQELQAFYKDLQETSQQFLSETPQAGIAQAVKQAQELLAFHKELQETSQQFLSATAQARIAYAEKQAQELLAFYQEVQETSQQFLSATAQARIAQAEKQAQELLAFHKELQETSQQFLSATADARTAQAKEQKESLLKFRQDLFVSIFG*
1013.606	1515.25864	MISLMAKIRQELLKLRNRRNLS*
1051.346	1323.88065	MISLMAKIRQEHQSIAEKVAELSLETRVFLSVTTAERQEQAKKQAQEPLAFHKELQEASQQFLSATADARTAQAKGQKESLLKFRQDLFVSIFG*
1126.825	916.269967	MISLMAKIRQEHQSIAEKVAELSLETREFLSVTTAKRQEQAEKQAQELLAFYQEVRETSQQFLSATAQARIAQAEKQAQELLAFYQEVRETSQQFLSATADARTAQAKEQKESLLKFRQDLFVSIFG*
1051.346	1141.03509	MISLMAKIRQEHQSIAEKVAELSLETREFLSLTTAKRQEQAEKQAQELLAFHKELQETSQQFLSATADARTARAKEQKESLLKFRQDLFVSIFG*
1126.825	868.099197	MISLMAKIRQEHQSIAKKVAELSLETGEFLSVTTAKRQEQAEKQAQELQAFYKDLQETSQQFLSETAQARIAQAEKQAQELLAFHKELQETSQQFLSATAQARIAQAEKQAQELLAFYQEVRDTSQQFLSATAQARIAQAEKQAQELLAFHKELQETSQQFLSATADARTAQAKEQKESLLKFRQDLFVSIFG*
1013.606	1497.15633	MISLMAKIRQEHQSIAEKVAELSLETREFLSLTTAKRQEQAEKQAQELLAFYKDLQETSQQFLSATADARTAQAKEQKEPLLKFRQDLFVSIFG*
1051.346	1084.96914	MISLMAKIRQEHQSIAEKVAELSLETREFLSVTTAKRQEQAEKQAQELLAFHKELQETSRQFLSASADARTAQAKEQKGSLLKFRQDLFVSIFG*
975.8665	1737.55641	MISLMAKIRQEHQSIAEKVAELSLETREFLSLTIAKRQEQAEKQAQELQAFYKDLQETSQQFLSATADARTAQAKEQKEPLLKFRQDLFVSIFG*
1126.825	948.192345	MISLMAKIRQEHQSIAKKVAELSLETGEFLSVTTAKRQEQAEKQAQELQAFYKDLQETSQQFLSETAQARIAQAEKQAQELLAFHKELQETSQQFLSATAQARIAQAEKQAQELLAFYQEVRDTSQQFLSATAQARIAQAEKQAQELLAFHKELQETSQQFLSATADXRTAQAKEQKESLLKFRQDLFVSIFG*
1013.606	901.228579	MISLMAKIRQEHQSIAEKVAELSLETREFLSVTTAKRQEQAEKQAQELQAFYKDLQETSQQFLSETAQARIAQAKEQKESLLKFRQDLFVSIFG*
975.8665	733.077641	MISLMAKIRQEHQSIAEKVAELSLETREFLSVTTAKRQEQAEKQAQELQAFYKDLQETSQQFLSETAQARIAQAEKQAQELLAFHKELQETSQQFLSATAQARIAQAKEQKESLLKFRQDLFVSIFG*
1051.346	1419.11456	MISLMAKIRQEHQSIAEKVAELSLETREFLSVTTAKRQEQAEKQAQEPQAFYKDLQETSQQFLSETAQARIAQAEKQAQELLAFHKELQETSQQFLSATAQARIAQAEKQAQELLAFYQEVRETSQQFLSATAQARIAQAEKQAQELLAFHKELQETSQQFLSATADARTAQAKEQKESLLKFRQDLFVSIFG*

1277.783	347.08149	MISLMAKIRQEHQSIAEKVAELSLETREFLSVTTAKRQEQAEKQAQELQAFYKDLQETSQQFLSETAQARIAQAEKQAQELLAFHKELQETSQQFLSATAEARIAQAERQAQELLAFYQEVRETSQQFLSATAQARIAQAEKQAQELLAFHKELQETSQQFLSATADARTAQAKEQKESLLKFRQDLFVSIFG*
1164.564	465.709117	MISLMAKIRQEHQSIAEKVAELSLETREFLSVTTAKRQEQAEKQAQELQAFYKDLQETSQQFLSETAQARIAQAEKQAQELLAFHKELQETSQQFLSATAQARIAQAEKQAQELLAFHKELQETSQQFLSATADARTAQAKEQEESLLKFRQDLFVSIFG*
1202.304	452.831946	MISLMAKIRQEHQSIAEKVAELSLETREFLSVTTAKRQEQAEKQAQELLAFYQEVRETSQQFLSATAQARIAQAEKQAQELLAFYQGVRETSQQFLSATAQARIAQAEKQAQELLAFHKELQETSQQFLSATADARTAQAKEQKESLLKFRQDLFVSIFG*
1240.043	572.437374	MISLMAKIRQEHQSIAEKVAELSLETREFLSVTTAKRQEQAEKRAQELQAFYKDLQETSQQFLSETAQARIAQAEKQAQELLAFHKELQETSQQFLSATAQARIAQAEKQAQELLAFYQEVRETSQQFLSATAQARIAQAKEQKESLLKFRQDLFVSIFG*
1202.304	385.817794	MISLMAKIRQEHQSIAEKVAELSLETREFLSVTTAKRQEQAEKQAQELLAFYQEVRETSQQFLSATAQARIAQAEKQAQELLAFYQEVRETSQQFLSATAQARIAQAEKQAQELLAFHKELQETSQQFLSATADARTAQAKEQKESLLKFRQDLFVSIFG*
1240.043	400.757309	MISLMAKIRQEHQSIAEKVAELSLETRELLSVTTAKRQEQAEKQAQELQAFYKDLQETSQQFLSETAQARIAQAEKQAQELLAFHKELQETSQQFLSATAQARIAQAEKQAQELLAFYQEVRETSQQFLSATAQARIAQAEKQAQELLAFHKELQETSQQFLSATADARTAQAKEQKESLLKFRQDLFVSIFG*
1315.522	340.046225	MISLMAKIRQEHQSIAEKVAELSLETREFLSVTTAKRQEQAEKQAQELQAFYKDLQETSQQFLSETAQARIAQAEKQAQELLAFHKELQETSQQFLSATAQARIAQAEKQAQELLAFYQEVRETSQQFYQQQPKQELLKLKYKLKNC*
1202.304	529.801883	MISLMAKIRQEHQSIAEKVAELSLETREFLSVTTAKRQEQAEKQAQELQAFYKDLQETSQQFLSETAQARIAQAEKQAQELLAFHKELQETSQQFLSATADARTAQAKEQKESLLKFRQDLFVSIFG*
1202.304	466.10704	MISLMAKIRQEHQSIAEKVAELSLETREFLSVTTAKRQEQAEKQAQELQAFYKDLQETSQQFLSATAQARIAQAEKQAQELLAFYQEVRETSQQFLSATAQARIAQAEKQAQELLAFHKELQETSQQFLSATADARTAQAKEQKESLLKFRQDLFVSIFG*
1202.304	443.538312	MISLMAKIRQEHQSIAEKVAELSLETREFLSVTTAKRQEQAEKQAQELLAFYKDLQETSQQFLSETAQARIAQAEKQAQELLAFYQELRETSQQFLSATAQARIAQAEKQAQELLAFYQEVRETSQQFLSATAHARIAQAEEXNE*
1202.304	453.177624	MISLMAKIRQEHQSIAEKVAELSLETREFLSVTTAKRQEQAEKQAQELQAFYKDLQETSQQFLSETAQARIAQAEKQAQELLAFHKELQETSQQFLSATAQARIAQAEKQAQELLAFYQEVRETSQQFLSATAQARIAQAEKQAQELQAFYKDLQETSQQFLSETAQARIAQAEKQAQELLAFHKELQETSQQFLSATAQARIAQAEKQAQELLAFYQEVRETSQQFLSATAQARIAQAEEQAQELLAFHKELQETXXXFLSXTADARTAQAXEQKESLLXXPXGIXLXSXLLXKLVLXX
1240.043	519.57938	MISLMAKIRQEHQSIAEKVAELSLETREFLSVTTAKRQEQAEKQAQELQAFYKDLQETSQQFLSATAQARIAQAEKQAQELLAFHKELQETSQQFLSATAQARIAQAEKQAQELLAFYQEVRETSQQFLSATAQARIAQAEKQAQELLAFHKELQETSQQFLSATADARTAQAKEQKESLLKFRQDLFVSIFG*
1240.043	377.069928	MISLMAKIRQEHQSIAEKVAELSLETREFLSVTTAKRQEQAEKQAQELQAFYKDLQETSQQFLSETAQARIAQAEKQAQELLAFHKELQETSQQFLSATAQARIAQAEKQAQELLAFYQEVRETSQQFLSATAQARIAQAEKQAQELQAFYKDLQETSQQFLSETAQARIAQAEKQAQELLAFHKELQETSQQFLSATAQARIAQAEKQAQELLAFYQEVRETSQQFLSATAQARIAQAEEQAQELLAFHKXNFXKQVSSFYQQQPTXELLKLXXEXISPEIXSGFVXEYXWXN*
1202.304	508.07192	MISLMAKIRQEHQSIAEKVAELSLETREFLSVTTAKRQEQAEKQAQELQAFYKDLQETSQQFLSETAQARNAQAEKQAQELLAFHKELQETSQQFLSATA

		QARIAQAEKQAQELLAFYQEVRETSQQFLSATAQARIAQAEKQAQELLAF HKELQETSQQFLSATADARTAQAKEQKESLLKFRQDLFVSIFG*
1202.304	427.641427	MISLMAKIRQEHQSIAEKVAELSLETREFLSVTTAKRQEQAEKQAQELQAF YKDLQETSQQFLSETAQARIAQAEKQAQELLAFHKELQETSQQFLSATAQ ARIAQAEKQAQELLAFYQEVRETSQQFLSATAQARIAQAEKQAQELQAFY KDLQETSQQFLSETAQARIAQAEKQAQELLAFHKELQETSQQFLSATAQA RIAQAEKQAQELLAFYQEVRETSQQFLSATAQARIAXXXXNKLKNC*
1164.564	596.16856	MISLMAKIRQEHQSIAEKVAELSLETREFLSVTTAKRQEQAEKQAQELQAF YKDLQETSQQFLSETAQARIAQAEKQAQELLAFHKELQETSQQFLSATAQ ARIAQAEKQAQELLAFYQEVRETSQQFLSATAQARIAQAEKQAQELQAFY KDLQETSQQFLSETAQARIAQAEKQAQELLAFHKELQETSQQFLSATAQA RIAQAEKQAQELLAFYQEVRETSQQFLSATAQARIAQAEEQAQELLAFHK NFXRXVXQXFFISNXRXXXCSAXGTXXSLXKFRQDLFVSILXXLVLXXXXX
1202.304	621.671708	MISLMAKIRQEHQSIAEKVAELSLETREFLSVTTAKRQEQAEKQAQELQAF YKDLQETSQQFLSETAQARIAQAEKQAQELLAFHKELQETSQQFLSATAQ ARIAQAEKQAQELLAFYQEVRETSQQFLSATAQARIAQAEKQAQELLAYH KELQETSQQFLSATADARTAQAKEQKESLLKFRQDLFVSIFG*
1202.304	410.682463	MISLMAKIRQEHQSIAEKVAELSLETREFLSVTTAKRREQAEKQAQELQAF YKDLQETSQQFLSETAQARIAQAEKQAQELLAFHKELQETSQQFLSATAD ARTAQAKEQKESLLKFRQDLFVSIFG*
1202.304	349.786962	MISLMAKIRQEHQSIAEKVAELSLETREFLSVTTAKRQEQAEKQAQELQAF YKDLQETSQQFLSATAQARIAQAEKQAQELIAFHKELQETSQQFLSATAD ARTAQAKEQKESLLKFRQDLFVSIFG*
824.9085	2733.93396	MISLMAKIRQEHQSIAEKVAELSLETREFLSVTTAKRQEQAERQAQELQAF YRDLQETSQQFLPETAQARIAQAEKQAQELLAFHKELQETSQQFLSATAQ ARIAQAEKQAQELLAFYQEVRETSQQFLSATAQARIAQAEKQAQELLAFH KELQETSQQFLSATADARTAQAKERKESLLKFRQDLFVSIFG*
938.127	2009.9254	MISLMAKIRQEHQSIAEKVAELSLETREFLSVTTAKRQEQAEKQAQELQAF YKDLQETSQQFLSETAQARIAQAEKQAQELLAFHKELQETSQQFLSETAQ ARIAQAEKQAQELLAFHKELQETSQQFLSATADARTAQAKEQKESLLKFR QDLFVSIFG*
711.69	2742.04099	MISLMAKIGKNISQ*
673.9505	2564.67277	MISLMAKIRQEHQSIAEKVAELSLETREFLSVTTAKRQEQAEKQAQELQAF YKDLQETSQQFLSETAQARIAQAEKQAQELLAFHKEPQETSQQFLSATAD ARTAQAKEQKESLLKFRQDLFVSIFG*
711.69	1890.16683	MISLMAKIRQEHQSIAEKVAELSLETREFLSVTTAKRQEQAEKQAQELQAF YKDLQETSQQFLSETAQARIAQAEKQAQELLAFHKELQETSQQFLSATAQ ARIAQAEKQAQELLAFYQEVRETSQQFLSATAQARIAQAEKQAQELQAFY KDLQETSQQFLSETAQARIAQAEKQAQELLAFHKELQETSQQFLSATAQA RIAQAEKTSARTVSILSRSSGNKSAVFISNSPXQELLXLKTSSRTVSIPQRT SRKQVSSFYQXQPTQELLS*
749.4295	950.088512	MISLMAKIRQEHQSIAEKVAELSLETREFSSVTTAKRQEQAEKQAQELQAF YKDLQETSQQFLSATAQARIAQAKEQKESLLKFRQDLFVSIFG*
1013.606	2063.29823	MISLMAKIRQEHQSIAEKVAELSLGTREFLSVTTAKRQEQAEEQAQEPLAF HKELQETSQQFLSATADARTAQAKEQKESLLKFRQDLFVSIFG*
1013.606	1639.09972	MISLMAKIRQEHQSIXEKVAELSLETRVFLSVTTAERQEQAKKQAQEPLAF HKELQEASQQFLSATADARTAQAKGQKESLLKFRQDLFVSIFG*
1013.606	520.723939	MISLMAKIRQEHQSIAEKVAELSLETREFLSVTTAKRQEQAEKQAQELQAF YKDLQETSQQFLSETAQARIAQAEKQAQELLAFHKELQETSQQFLSATAQ ARIAQAEKQAQELLAFYQEVRETSQQFLSATAQARIAQAERQAQELQAFY KDLQETSQQFLSETAQARIAQAEKQAQELLAFHKELQETSQQFLSATAQA RIAQAEKQAQELLAFYQEVRETSQQFLSATAQARIXQAE*
711.69	785.779405	MISLMAKIRQEHQSIAEKVAELSLETREFLSVTTAKRQEQAEKQAQELQAF YKDLQETSQQFLSETAQARIAQAEKQAQELLAFHKELQETSQQFLPATAQ ARIAQAEKQAQGLLAFY*

1126.825	375.114594	MISLMAKIWQEHQSIAEKVAELSLETREFMSVTTAKRQEQAEKQAQELQAFYKDLQETSQQFLSETAQARIAQAEKQAQELLAFHKELQETSQQFLSATAQARIA*
1391.001	327.491186	MISLMAKIRQEHQSIAEKVAELSLETREFLSVTTAKRQEQAEKQAQELQAFYKDLQETSQ*
1051.346	474.951586	MISLMAKIRQEHQSIAEKVAELSLETREFLSVTTAKRQEQAEKQAQELQAFYKDLQETSQQFLSETAQARIAQAEKQAQELLAFHKELQETSQQFLSATAQARIAQAEKQAQELLAFYQEVRETSQQFLSATAQARIAQAEKQAQELQAFYKDLQETSQQFLSETAQARIAQAEKQAQELLAFHKELQETSQQFLSATAQARIAQAEKQAQELLAFYXRKFGKTSQQFLSATAQARXCSS*
1315.522	403.151717	MISLMAKIRQEHQSIAEKVAELSLETREFLSVTTAKRQEQAEKQAQELQAFYKDLQETSQQFLSETAQARIAQAEKQAQELLAFHKELQETSQQFLSATAQARIAQAEKQAQELLAFYQEVRETSQQFLSATAQARIAQAEKQAQELLAFHKELQETSQQFLSATADARTAQAKEQKESLLKFRQDLFVSIFG*
1466.48	313.652835	MISLMAKIRQEHQSIAEKVAELSLETREFLSVTTAKRQEQAEKQAQELQALYKDLQETSQQFLSETAQARIAQAEKQAQELLAFHKELQETSQQFLSATAQARIAQAEKQAQELLAFYQEVRETSQQFLSATAQARIAQAEKQAQELLAFHKELQETSQQFLSATADARTAQAKEQKESLLKFRQDLFVSIFG*
1164.564	1164.564	MISLMAKIRQEHQSIAEKVAELSLETREFLSVTTAKRQEQAEKQAQELQAFYKDLQETSQQFLSATADARTAQAKEQKESLLKFRQDLFVSIFG*
711.69	1496.97584	MISLMAKIRQEHQSIAEKVAELSLETREFLSVTTAKRQEQAEKQAQELQAFYKDLQETSQQFLSETAQARIAQAEKQAQELLAFHKELQETSQQFLSATAQARIAQAEKQAQELLAFYQEVRETSQQFLSATAQARIAQAEKQAQELLAFHKELQETSQQFLSATADARTAQAKEQKESLLKFRQDLFGVSLVKLVLSAPGSESSVVLRSEDPLALGSEDSLVPDXKGSLAPXSEQXALPEIVQTTAPTLPTTPXXDGF*
711.69	2330.93592	MISLMAKIRQEHQSIAEKVAELSLETREFLSVTTAKRQEQAEKQAQELQAFYKDLQETSQQFLSETAQARIAQAEKQAQELLAFHKELQETSQQFLSATAQARIAQAEKQAQELLAFYQEVRETSQQFLSATAQARIAQAEKQAQELLAFHKELQETSQQFLSXTADXXTAQAKEQKESLLXFRXDLIVSIXG*
711.69	2703.52291	MIL*
673.9505	1512.81543	MISLMAKIRQEHQSIAEKVAELSLETREFLSVTTAKRQEQAEKQAQELQAFYKDLQETSQQFLSETAQARIAQAEKQAQELLAFHKELQETSQQFLSATADARTAQAKEQKESLLKFRQDLFVSSLVKLVLSAPGSESSVVLRSEDPLALGSEDSLVPDSKGSLAPSSEQPALPEIVQTTAPTLPTTPPATVFEKKNVAEHTVDXTN*
673.9505	3109.40905	MISLMAKIRQEHQSIAEKVAELSLETREFLSVTTAKRQEQAEKQAQELQAFYKDLQETSQQFLSATAQARIAQAEKQAQELLAFHKELQETSQQFLSATAQARIAQAEKQAQELLAFYQEVRETSQQFLSETAQARIAQAEKQAQELLAFHKELQETSQQFLSATADARTAQAKEQKESLLKFRQDLFVSIFG*
636.211	3016.167	MISLMAKIRQEHQSIAEKVAELSLETREFLSVTTAKRQEQAEKQAQELQAFYKDLQETSQQFLSETAQARIAQAEKQAQELLAFHKELQETSQQFLSXTADARTAQAXEQKESLLKFRQDLFVXIFG*
598.4715	4715.74587	MISLMAKIRQEHQSIAEKVAELSLETREFLSVTTAKRQEQAEKQAQELQAFYKDLQETSQQFLSETAQARIAQAEKQAQELLAFHKELQETSQQFLSATAQARIAQAEKQAQELLAFYQEVRETSQQFLSATAQARIAQAEKQAQELLAFHKELQETSQQFLSATAQARIAQAEKQAQELLAFYQEVRETSQQFLSATAQARIAQAEKQAQELLAFHKELQETSQQFLSATADARTAQAKEQXESLLKFRQDLFVSIFG*
673.9505	2482.16933	MISLMAKIRQEHQSIAEKVAELSLETREFLSVTTAKRQEQAEKQAQELQAFYKDLQETSQQFLSETAQARIAQAEKQAQELLAFHKELQETSQQFLSETAQARIAQAEKQAQELLAFHKELQETSQQFLSATAQARIAQAEKQAQELLAFYQEVRETSQQFLSATAQARIAQAEKQAQELLAFHKELQETSQQFLSATADARTAQAKEQKESLLKFRQDLFVSIFG*

560.732	4641.95945	MISLMAKIRQEHQSIAEKVAELSLETREFLSVTTAKRQEQAEKQAQELQAFYKDLQETSQQFLSETAQARIAQAEKQAQELLAFHKELQETSQQFLSATAQARIAQAEKQAQELLAFYQEVRETSQQFLSATAQARIAQAEKQAQELLAFHKELQETSQQFLSATADARTAQAKEXKESLLKFRQDLFVSIFG*
673.9505	2092.58558	MISLMAKIRQEHQSIAEKVAELSLETREFLSVTTAKRQEQAEKQAQELRAFYKDLQETSQQFLSATADARTAQAKEQKESLLKFRQDLFVSIFG*
749.4295	952.369412	MISLMAKIRQEHQSIAEKVAELSLETREFLSVTTAKRQEQAEKQAQELLAFYQEVRETSQQFLSATAQARIAQAEKQAQELLAFHKELQETSQQFLSATAQARIAQAEKQAQELLAFYQEVRETSQQFLSATAQARIAQAEKQAQELLAFHKELQETSQQFLSATADARTAQAKEQKESLLKFRQDLFVSIFG*
598.4715	2253.16403	MISLMAKIRQEHQSIAEKVAELSLETREFLSVTTAKRQEQAEKQAQELLAFHKELQETSQQFLSATAQARIAQAEKQAQELLAFYQEVRETSQQFLSATAQARIAQAEKQAQELLAFYQEVRETSQQFLSATAQARIAQAEKQAQELLAFHKEPQETSQQFLSATADARTAQAKEQKESLLKFRQDLFVSIFG*
560.732	3527.49429	MISLMAKIRQEHQSIAEKVAELSLETREFLSVTTAKRQEQAEKQAQELQAFYKDLQETSQQFLSATADARTAQAKEQKEXLLKFRQDLFVSIFG*
598.4715	3533.32126	MIF*
485.253	7356.95946	MISLMAKIRQEHQSIAEKVAELSLETREFLSVTTAKRQEQAEKQAQELQAFYKDLQETSQQFLSETAQARIAQAEKQAQELLAFHKELQETSQQFLSATAQARIAQAEKQAQELLAFHKELQETSQQFLSATAQARIAQAEKQAQELLAFYQEVRETSQQFLSATAQARIAQAEKQAQELLAFHKELQETSQQFLSATADAXTAQAKEQXESLLKFRQDLFVSIFGXN*
824.9085	544.555465	MISLMAKIRQEHQSIAEKVAELSLETREFLSVTTAKRQEQAEKQAQELQAFYKDLQETSQQFLSETAQARIAQAEKQAQELLAFHKELQETSQQFLSATAQARIAQAEKQAQELLAFHKELQETSQQFLSATADARTAQAKEQKESLLKFRQDLFVSIFG*
673.9505	3199.89403	MISLMAKIRQEHQSIAEKVAELSLETREFLSVTTAKRQEQAEKQAQELQAFYKELQETSQQFLSATAQARIAQAEKQAQELLAFYQEVRETSQQFLSATAQARIAQAEKQAQELLAFYQEVRETSQQFLSATAQARIAQAEKQAQELLAFYQEVRETSQQFLSATADARTAQAKEQKESLLKFRQDLFVSIFX*
673.9505	1791.36115	MISLMAKIRQEHQSIAEKVAELSLETREFLSVTTAKSQEQAEKQAQELQAFYKDLQETSQQFLSETAQARIAQAEKQAQELLAFYQEVRETSQQFLSATAQARIAQAEKQAQELLAFHKELQETSQQFLSATADARTAQAKEQKESLLKFRQDLFVXIFG*
598.4715	4729.16152	MISLMAKIRQEHQSIAEKVAELSLETREFLSVTTAKRQEQAEKQAQELQAFYKDLQETSQQFLSETAQARIAQAEKQAQELLAFHKELQETSQQFLSATAQARIAQAEKQAQELLAFYQEVRETSQQFLSATAQARIAQAEKQAQELLAFHKELQETSQQFLSATADARTAQAKGQKESLLKFRQDLFVSIFG*
711.69	1446.68824	MISLMAKIRQEHQSIAEKVAELSLETREFLSVTTAKRQEQAEKQAQELLAFHKELQETSQQFLSATAQARIAQAEKQAQELLAFYQEVRETSQQFLSATADARTAQAKEQKESLLKFRQDLFVSIFG*
1013.606	1923.09804	MISLMAKIRQEHQSIAEKVAELSLETREFLSVTTAKRQEQAEKQAQELQAFYKDLQETSQQFLSETAQARIAQAGKQAQELLAFHKELQETSQQFLSATADARTAQAKEQKESLLKFRQDLFVSIFG*
711.69	800.073878	MISLMAKIRQEHQSIAEKVAELPLETREFLSVTTAKRQEQAEKQAQELQAFYKDLQETSQQFLSETADARTAQAKEQKESLLKFRQDLFVSIFG*
636.211	1300.78639	MISLMAKIRQEHQSIAEKVAELSLETREFLPVTTAKRQEQAEKQAQELQAFYKDLQETSQQFLSATADARTAQAKEQKESLLKFRQDLFVSIFG*
711.69	2144.35028	MISLMAKIRQEHQSMAEKVAELSLETREFLSVTTAKRQEQAEKQAQELQAFYKDPQETSQQFLSETAQARIAQAEKQAQELLAFHKELQETSQQFLSATADARTAQAKEQKESLLKFRQDLFVSIFG*
711.69	2414.473	MISLWQKSGKNISQ*

Supplementary Table T3. The Multiplexing Matrix, *M*, for linear unmixing of acoustic reporter signals.

Pressure (kPa)	Reference $bARG_{560}$	Reference $bARG_{710}$
409.774	27.63151	27.95981
447.5135	31.04515	29.0257
485.253	37.22215	28.64597
522.9925	46.85362	30.12973
560.732	66.07532	32.17465
598.4715	93.75065	35.40891
636.211	128.2014	38.9426
673.9505	175.7886	47.68787
711.69	236.9551	59.55929
749.4295	313.9119	74.73107
787.169	406.6985	102.7811
824.9085	503.6675	142.5782
862.648	609.3632	194.9001
900.3875	718.8943	264.3136
938.127	830.8023	348.3042
975.8665	948.9999	442.6345
1013.606	1062.433	546.5271
1051.346	1169.691	663.8833
1089.085	1278.423	796.4225
1126.825	1376.163	921.949
1164.564	1476.647	1050.949
1202.304	1570.075	1181.289
1240.043	1660.065	1306.391
