# Supplementary material for: Multiplexed ultrasound imaging of gene expression
Source: Nat Methods. 2025 Nov 18;22(12):2594–600. doi: 10.1038/s41592-025-02825-w (PMC12695634; doi:10.1038/s41592-025-02825-w)
Supplement: Supplementary file 2 — Reporting Summary [file 41592_2025_2825_MOESM2_ESM.pdf]

nature portfolio

Corresponding author(s): Mikhail Shapiro

Last updated by author(s): Dec 6, 2024

Reporting Summary

Nature Portfolio wishes to improve the reproducibility of the work that we publish. This form provides structure for consistency and transparency in reporting. For further information on Nature Portfolio policies, see our Editorial Policies and the Editorial Policy Checklist.

Statistics

For all statistical analyses, confirm that the following items are present in the figure legend, table legend, main text, or Methods section.

n/a	Confirmed	
☐	☒	The exact sample size (*n*) for each experimental group/condition, given as a discrete number and unit of measurement
☐	☒	A statement on whether measurements were taken from distinct samples or whether the same sample was measured repeatedly
☐	☒	The statistical test(s) used AND whether they are one- or two-sided<br>*Only common tests should be described solely by name; describe more complex techniques in the Methods section.*
☒	☐	A description of all covariates tested
☒	☐	A description of any assumptions or corrections, such as tests of normality and adjustment for multiple comparisons
☐	☒	A full description of the statistical parameters including central tendency (e.g. means) or other basic estimates (e.g. regression coefficient) AND variation (e.g. standard deviation) or associated estimates of uncertainty (e.g. confidence intervals)
☐	☒	For null hypothesis testing, the test statistic (e.g. *F*, *t*, *r*) with confidence intervals, effect sizes, degrees of freedom and *P* value noted<br>*Give P values as exact values whenever suitable.*
☒	☐	For Bayesian analysis, information on the choice of priors and Markov chain Monte Carlo settings
☒	☐	For hierarchical and complex designs, identification of the appropriate level for tests and full reporting of outcomes
☒	☐	Estimates of effect sizes (e.g. Cohen's *d*, Pearson's *r*), indicating how they were calculated

Our web collection on statistics for biologists contains articles on many of the points above.

Software and code

Policy information about availability of computer code

Data collection	We used MATLAB (Version 2021a, Mathworks) custom scripts, with functions provided by the Vantage 4.6.2 system (Verasonics), to acquire ultrasound images.
Data analysis	We used MATLAB (Version 2021a and later, Mathworks) custom scripts and Prism (Version 9 and later, Graphpad) for all data processing and statistical analysis. Illustrations were made in Affinity Designer (Version 2.3.5, Serif Europe).

For manuscripts utilizing custom algorithms or software that are central to the research but not yet described in published literature, software must be made available to editors and reviewers. We strongly encourage code deposition in a community repository (e.g. GitHub). See the Nature Portfolio guidelines for submitting code & software for further information.

Data

Policy information about availability of data

All manuscripts must include a data availability statement. This statement should provide the following information, where applicable:

- Accession codes, unique identifiers, or web links for publicly available datasets
- A description of any restrictions on data availability
- For clinical datasets or third party data, please ensure that the statement adheres to our policy

Processing scripts used to generate key figures and results will be posted to https://github.com/shapiro-lab. Raw data can be made available by M.G.S. upon reasonable request.

Human research participants

Policy information about studies involving human research participants and Sex and Gender in Research.

Reporting on sex and gender	No human research participants were used in this study.
Population characteristics	No human research participants were used in this study.
Recruitment	No human research participants were used in this study.
Ethics oversight	No human research participants were used in this study.

Note that full information on the approval of the study protocol must also be provided in the manuscript.

Field-specific reporting

Please select the one below that is the best fit for your research. If you are not sure, read the appropriate sections before making your selection.

☒ Life sciences ☐ Behavioural & social sciences ☐ Ecological, evolutionary & environmental sciences

For a reference copy of the document with all sections, see nature.com/documents/nr-reporting-summary-flat.pdf

Life sciences study design

All studies must disclose on these points even when the disclosure is negative.

Sample size	The numbers of biological replicates were chosen based on preliminary experiments. No statistical tests were used to predetermine sample size for statistical significance.
Data exclusions	Cell cultures that exhibited no or minimal gas vesicle expression (determined by no/low patch opacity or no/low destructive ultrasound signals) were excluded. Where relevant for imaging bARG710, cell cultures that exhibited no or minimal red fluorescent protein expression (indicative of no or minimal gvpC-L154P co-expression) were excluded.
Replication	Replicates are reported in-text or in figure legends.
Randomization	Animals were randomly distributed into cages and ear-punched by animal care staff. Cages of animals were randomly chosen for different acoustic color conditions (bARG560, bARG710, or a 1:1 mixture). In all other experiments, samples were allocated randomly.
Blinding	Blinding was not applicable to our study because our experiments did not involve human participants and all data collection, processing and analysis methods were quantitative and identical across experimental groups.

Reporting for specific materials, systems and methods

We require information from authors about some types of materials, experimental systems and methods used in many studies. Here, indicate whether each material, system or method listed is relevant to your study. If you are not sure if a list item applies to your research, read the appropriate section before selecting a response.

Materials & experimental systems

n/a	Involved in the study	
☒	☐	Antibodies
☐	☒	Eukaryotic cell lines
☒	☐	Palaeontology and archaeology
☐	☒	Animals and other organisms
☒	☐	Clinical data
☒	☐	Dual use research of concern

Methods

n/a	Involved in the study	
☒	☐	ChIP-seq
☒	☐	Flow cytometry
☒	☐	MRI-based neuroimaging

Eukaryotic cell lines

Policy information about cell lines and Sex and Gender in Research

Cell line source(s)	MC26 cells were ordered from BioHippo (Cat. 400156).

Authentication	The cells were authenticated by BioHippo before delivery using short tandem repeat (STR) profiling.
Mycoplasma contamination	The cells were certified not contaminated by BioHippo and tested for mycoplasma contamination by our lab management team.
Commonly misidentified lines (See ICLAC register)	No commonly misidentified lines were used in this study.

Animals and other research organisms

Policy information about studies involving animals; ARRIVE guidelines recommended for reporting animal research, and Sex and Gender in Research

Laboratory animals	Female BALB/c mice (Strain #000651, Jackson Laboratory) aged 5-10 weeks were used for all animal experiments. Animals were housed in a facility maintained at 21–24°C and 30–70% humidity, with a lighting cycle of 13 hours on (6:00–19:00) and 11 hours off. Mice were anesthetized with 1–3% isoflurane in 100% oxygen using a nose cone co-linked to a vacuum line for active scavenging, and kept warm on a heated stage during procedures.
Wild animals	This study did not involve wild animals.
Reporting on sex	This study does not apply to one sex, sex was not considered in the study design, and no methods were used for assigning sex. Female mice were used because the MC26 murine colon carcinoma cell line was derived from a female BALB/c mouse and we sought to achieve a syngeneic mouse model.
Field-collected samples	This study did not involve samples collected from the field.
Ethics oversight	All animal protocols were approved by the Institutional Animal Care and Use Committee (IACUC) at the California Institute of Technology (Protocol #1735) and comply with federal and state regulations governing the humane care and use of laboratory animals.

Note that full information on the approval of the study protocol must also be provided in the manuscript.
